# Supplementary material for: A Critical View of the Application of the APEX Software (Aqueous Photochemistry of Environmentally-Occurring Xenobiotics) to Predict Photoreaction Kinetics in Surface Freshwaters
Source: Molecules. 2019 Dec 18;25(1):9. doi: 10.3390/molecules25010009 (PMC7017383; doi:10.3390/molecules25010009)
Supplement: Supplementary file 1 [file molecules-25-00009-s001.zip › 3.2.4_gcc-4.4.0.zip!/3.2.4_gcc-4.4.0/3.2.4_gcc-4.4.0/share/octave/packages/gsl-1.0.8/doc/gsl_sf.pdf]

Øgsl˙sf -*- texinfo -*-

`gsl_sf ()` [Loadable Function]

Octave bindings to the GNU Scientific Library. All GSL functions can be called with by the GSL names within octave.

Øclausen -*- texinfo -*-

y `= clausen (`*x*`)` [Loadable Function]

`[`*y*`,` *err*`] = clausen (...)` [Loadable Function]

The Clausen function is defined by the following integral,

Cl˙2(x) = - int˙0^x dt log(2 sin(t/2))

It is related to the dilogarithm by Cl˙2(theta) = Im Li˙2(exp(i theta)).

err contains an estimate of the absolute error in the value *y*.

This function is from the GNU Scientific Library, see `http://www.gnu.org/software/gsl/` for documentation.

Ødawson -*- texinfo -*-

y `= dawson (`*x*`)` [Loadable Function]

`[`*y*`,` *err*`] = dawson (...)` [Loadable Function]

The Dawson integral is defined by exp(-x^2) int˙0^x dt exp(t^2). A table of Dawson integral can be found in Abramowitz & Stegun, Table 7.5.

err contains an estimate of the absolute error in the value *y*.

This function is from the GNU Scientific Library, see `http://www.gnu.org/software/gsl/` for documentation.

Ødebye˙1 -*- texinfo -*-

y `= debye_1 (`*x*`)` [Loadable Function]

`[`*y*`,` *err*`] = debye_1 (...)` [Loadable Function]

The Debye functions are defined by the integral

D˙n(x) = n/x^n int˙0^x dt (t^n/(e^t - 1)).

For further information see Abramowitz & Stegun, Section 27.1.

err contains an estimate of the absolute error in the value *y*.

This function is from the GNU Scientific Library, see `http://www.gnu.org/software/gsl/` for documentation.

Ødebye˙2 -*- texinfo -*-

y `= debye_2 (`*x*`)` [Loadable Function]

`[`*y*`,` *err*`] = debye_2 (...)` [Loadable Function]

The Debye functions are defined by the integral

D˙n(x) = n/x^n int˙0^x dt (t^n/(e^t - 1)).

For further information see Abramowitz & Stegun, Section 27.1.

err contains an estimate of the absolute error in the value *y*.

This function is from the GNU Scientific Library, see `http://www.gnu.org/software/gsl/` for documentation.

Ødebye˙3 -*- texinfo -*-

`y = debye_3 (x)` [Loadable Function]
`[y, err] = debye_3 (...)` [Loadable Function]

The Debye functions are defined by the integral

D˙n(x) = n/x^n int˙0^x dt (t^n/(e^t - 1)).

For further information see Abramowitz & Stegun, Section 27.1.

err contains an estimate of the absolute error in the value *y*.

This function is from the GNU Scientific Library, see http://www.gnu.org/software/gsl/ for documentation.

Ødebye˙4 -*- texinfo -*-

`y = debye_4 (x)` [Loadable Function]
`[y, err] = debye_4 (...)` [Loadable Function]

The Debye functions are defined by the integral

D˙n(x) = n/x^n int˙0^x dt (t^n/(e^t - 1)).

For further information see Abramowitz & Stegun, Section 27.1.

err contains an estimate of the absolute error in the value *y*.

This function is from the GNU Scientific Library, see http://www.gnu.org/software/gsl/ for documentation.

Øerf˙gsl -*- texinfo -*-

`y = erf_gsl (x)` [Loadable Function]
`[y, err] = erf_gsl (...)` [Loadable Function]

These routines compute the error function erf(x) = (2/sqrt(pi)) int˙0^x dt exp(-t^2).

err contains an estimate of the absolute error in the value *y*.

This function is from the GNU Scientific Library, see http://www.gnu.org/software/gsl/ for documentation.

Øerfc˙gsl -*- texinfo -*-

`y = erfc_gsl (x)` [Loadable Function]
`[y, err] = erfc_gsl (...)` [Loadable Function]

These routines compute the complementary error function erfc(x) = 1 - erf(x) = (2/sqrt(pi)) int˙x^infty exp(-t^2).

err contains an estimate of the absolute error in the value *y*.

This function is from the GNU Scientific Library, see http://www.gnu.org/software/gsl/ for documentation.

Ølog˙erfc -*- texinfo -*-

`y = log_erfc (x)` [Loadable Function]
`[y, err] = log_erfc (...)` [Loadable Function]

These routines compute the logarithm of the complementary error function log(erfc(x)).

err contains an estimate of the absolute error in the value *y*.

This function is from the GNU Scientific Library, see http://www.gnu.org/software/gsl/ for documentation.

Øerf˙Z -*- texinfo -*-

`y = erf_Z (x)` [Loadable Function]
`[y, err] = erf_Z (...)` [Loadable Function]

These routines compute the Gaussian probability function Z(x) = (1/(2pi)) exp(-x^2/2).

err contains an estimate of the absolute error in the value *y*.

This function is from the GNU Scientific Library, see http://www.gnu.org/software/gsl/ for documentation.

Øerf˙Q -*- texinfo -*-

`y = erf_Q (x)` [Loadable Function]
`[y, err] = erf_Q (...)` [Loadable Function]

These routines compute the upper tail of the Gaussian probability function Q(x) = (1/(2pi)) int˙x^infty dt exp(-t^2/2).

err contains an estimate of the absolute error in the value *y*.

This function is from the GNU Scientific Library, see http://www.gnu.org/software/gsl/ for documentation.

Øhazard -*- texinfo -*-

`y = hazard (x)` [Loadable Function]
`[y, err] = hazard (...)` [Loadable Function]

The hazard function for the normal distrbution, also known as the inverse Mill's ratio, is defined as h(x) = Z(x)/Q(x) = sqrt{2/pi exp(-x^2 / 2) / erfc(x/sqrt 2)}. It decreases rapidly as x approaches -infty and asymptotes to h(x) sim x as x approaches +infty.

err contains an estimate of the absolute error in the value *y*.

This function is from the GNU Scientific Library, see http://www.gnu.org/software/gsl/ for documentation.

Øexpm1 -*- texinfo -*-

`y = expm1 (x)` [Loadable Function]
`[y, err] = expm1 (...)` [Loadable Function]

These routines compute the quantity exp(x)-1 using an algorithm that is accurate for small x.

err contains an estimate of the absolute error in the value *y*.

This function is from the GNU Scientific Library, see http://www.gnu.org/software/gsl/ for documentation.

Øexprel -*- texinfo -*-

`y = exprel (x)` [Loadable Function]
`[y, err] = exprel (...)` [Loadable Function]

These routines compute the quantity (exp(x)-1)/x using an algorithm that is accurate for small x. For small x the algorithm is based on the expansion (exp(x)-1)/x = 1 + x/2 + x^2/(2*3) + x^3/(2*3*4) + dots.

err contains an estimate of the absolute error in the value *y*.

This function is from the GNU Scientific Library, see http://www.gnu.org/software/gsl/ for documentation.

Øexprel˙2 -*- texinfo -*-

`y = exprel_2 (x)` [Loadable Function]
`[y, err] = exprel_2 (...)` [Loadable Function]

These routines compute the quantity 2(exp(x)-1-x)/x^2 using an algorithm that is accurate for small x. For small x the algorithm is based on the expansion 2(exp(x)-1-x)/x^2 = 1 + x/3 + x^2/(3*4) + x^3/(3*4*5) + dots.

err contains an estimate of the absolute error in the value *y*.

This function is from the GNU Scientific Library, see http://www.gnu.org/software/gsl/ for documentation.

Øexpint˙E1 -*- texinfo -*-

`y = expint_E1 (x)` [Loadable Function]
`[y, err] = expint_E1 (...)` [Loadable Function]

These routines compute the exponential integral E˙1(x),

E˙1(x) := Re int˙1^infty dt exp(-xt)/t.

err contains an estimate of the absolute error in the value *y*.

This function is from the GNU Scientific Library, see http://www.gnu.org/software/gsl/ for documentation.

Øexpint˙E2 -*- texinfo -*-

`y = expint_E2 (x)` [Loadable Function]
`[y, err] = expint_E2 (...)` [Loadable Function]

These routines compute the second-order exponential integral E˙2(x),

E˙2(x) := Re int˙1^infty dt exp(-xt)/t^2.

err contains an estimate of the absolute error in the value *y*.

This function is from the GNU Scientific Library, see http://www.gnu.org/software/gsl/ for documentation.

Øexpint˙Ei -*- texinfo -*-

`y = expint_Ei (x)` [Loadable Function]
`[y, err] = expint_Ei (...)` [Loadable Function]

These routines compute the exponential integral E˙i(x),

Ei(x) := - PV(int˙{-x}^infty dt exp(-t)/t)

where PV denotes the principal value of the integral.

err contains an estimate of the absolute error in the value *y*.

This function is from the GNU Scientific Library, see http://www.gnu.org/software/gsl/ for documentation.

ØShi -*- texinfo -*-

y = Shi (*x*) [Loadable Function]
[*y*, *err*] = Shi (...) [Loadable Function]

These routines compute the integral Shi(x) = int˙0ˆx dt sinh(t)/t.

err contains an estimate of the absolute error in the value *y*.

This function is from the GNU Scientific Library, see http://www.gnu.org/software/gsl/ for documentation.

ØChi -*- texinfo -*-

y = Chi (*x*) [Loadable Function]
[*y*, *err*] = Chi (...) [Loadable Function]

These routines compute the integral

Chi(x) := Re[gamma˙E + log(x) + int˙0ˆx dt (cosh[t]-1)/t] ,

where gamma˙E is the Euler constant.

err contains an estimate of the absolute error in the value *y*.

This function is from the GNU Scientific Library, see http://www.gnu.org/software/gsl/ for documentation.

Øexpint˙3 -*- texinfo -*-

y = expint_3 (*x*) [Loadable Function]
[*y*, *err*] = expint_3 (...) [Loadable Function]

These routines compute the exponential integral Ei˙3(x) = int˙0ˆx dt exp(-tˆ3) for x >= 0.

err contains an estimate of the absolute error in the value *y*.

This function is from the GNU Scientific Library, see http://www.gnu.org/software/gsl/ for documentation.

ØSi -*- texinfo -*-

y = Si (*x*) [Loadable Function]
[*y*, *err*] = Si (...) [Loadable Function]

These routines compute the Sine integral Si(x) = int˙0ˆx dt sin(t)/t.

err contains an estimate of the absolute error in the value *y*.

This function is from the GNU Scientific Library, see http://www.gnu.org/software/gsl/ for documentation.

ØCi -*- texinfo -*-

`y = Ci (x)` [Loadable Function]
`[y, err] = Ci (...)` [Loadable Function]

These routines compute the Cosine integral Ci(x) = -int˙x^infty dt cos(t)/t for x > 0.

err contains an estimate of the absolute error in the value *y*.

This function is from the GNU Scientific Library, see http://www.gnu.org/software/gsl/ for documentation.

Øatanint -*- texinfo -*-

`y = atanint (x)` [Loadable Function]
`[y, err] = atanint (...)` [Loadable Function]

These routines compute the Arctangent integral AtanInt(x) = int˙0^x dt arctan(t)/t.

err contains an estimate of the absolute error in the value *y*.

This function is from the GNU Scientific Library, see http://www.gnu.org/software/gsl/ for documentation.

Øfermi˙dirac˙mhalf -*- texinfo -*-

`y = fermi_dirac_mhalf (x)` [Loadable Function]
`[y, err] = fermi_dirac_mhalf (...)` [Loadable Function]

These routines compute the complete Fermi-Dirac integral F˙{-1/2}(x).

err contains an estimate of the absolute error in the value *y*.

This function is from the GNU Scientific Library, see http://www.gnu.org/software/gsl/ for documentation.

Øfermi˙dirac˙half -*- texinfo -*-

`y = fermi_dirac_half (x)` [Loadable Function]
`[y, err] = fermi_dirac_half (...)` [Loadable Function]

These routines compute the complete Fermi-Dirac integral F˙{1/2}(x).

err contains an estimate of the absolute error in the value *y*.

This function is from the GNU Scientific Library, see http://www.gnu.org/software/gsl/ for documentation.

Øfermi˙dirac˙3half -*- texinfo -*-

`y = fermi_dirac_3half (x)` [Loadable Function]
`[y, err] = fermi_dirac_3half (...)` [Loadable Function]

These routines compute the complete Fermi-Dirac integral F˙{3/2}(x).

err contains an estimate of the absolute error in the value *y*.

This function is from the GNU Scientific Library, see http://www.gnu.org/software/gsl/ for documentation.

Øgamma˙gsl -*- texinfo -*-

y = gamma_gsl (*x*) [Loadable Function]
[*y*, *err*] = gamma_gsl (...) [Loadable Function]

These routines compute the Gamma function Gamma(x), subject to x not being a negative integer. The function is computed using the real Lanczos method. The maximum value of x such that Gamma(x) is not considered an overflow is given by the macro GSL˙SF˙GAMMA˙XMAX and is 171.0.

err contains an estimate of the absolute error in the value *y*.

This function is from the GNU Scientific Library, see http://www.gnu.org/software/gsl/ for documentation.

Ølngamma˙gsl -*- texinfo -*-

y = lngamma_gsl (*x*) [Loadable Function]
[*y*, *err*] = lngamma_gsl (...) [Loadable Function]

These routines compute the logarithm of the Gamma function, log(Gamma(x)), subject to x not a being negative integer. For x<0 the real part of log(Gamma(x)) is returned, which is equivalent to log(|Gamma(x)|). The function is computed using the real Lanczos method.

err contains an estimate of the absolute error in the value *y*.

This function is from the GNU Scientific Library, see http://www.gnu.org/software/gsl/ for documentation.

Øgammastar -*- texinfo -*-

y = gammastar (*x*) [Loadable Function]
[*y*, *err*] = gammastar (...) [Loadable Function]

These routines compute the regulated Gamma Function Gamma^*(x) for x > 0. The regulated gamma function is given by,

Gamma^*(x) = Gamma(x)/(sqrt{2pi} x^{(x-1/2)} exp(-x)) = (1 + (1/12x) + ...) for x to infty

and is a useful suggestion of Temme.

err contains an estimate of the absolute error in the value *y*.

This function is from the GNU Scientific Library, see http://www.gnu.org/software/gsl/ for documentation.

Øgammainv˙gsl -*- texinfo -*-

y = gammainv_gsl (*x*) [Loadable Function]
[*y*, *err*] = gammainv_gsl (...) [Loadable Function]

These routines compute the reciprocal of the gamma function, 1/Gamma(x) using the real Lanczos method.

err contains an estimate of the absolute error in the value *y*.

This function is from the GNU Scientific Library, see http://www.gnu.org/software/gsl/ for documentation.

Ølambert˙W0 -*- texinfo -*-

`y = lambert_W0 (x)` [Loadable Function]
`[y, err] = lambert_W0 (...)` [Loadable Function]

These compute the principal branch of the Lambert W function, W˙0(x).

Lambert's W functions, W(x), are defined to be solutions of the equation W(x) exp(W(x)) = x. This function has multiple branches for x < 0; however, it has only two real-valued branches. We define W˙0(x) to be the principal branch, where W > -1 for x < 0, and W˙{-1}(x) to be the other real branch, where W < -1 for x < 0.

err contains an estimate of the absolute error in the value *y*.

This function is from the GNU Scientific Library, see http://www.gnu.org/software/gsl/ for documentation.

Ølambert˙Wm1 -*- texinfo -*-

`y = lambert_Wm1 (x)` [Loadable Function]
`[y, err] = lambert_Wm1 (...)` [Loadable Function]

These compute the secondary real-valued branch of the Lambert W function, W˙{-1}(x).

Lambert's W functions, W(x), are defined to be solutions of the equation W(x) exp(W(x)) = x. This function has multiple branches for x < 0; however, it has only two real-valued branches. We define W˙0(x) to be the principal branch, where W > -1 for x < 0, and W˙{-1}(x) to be the other real branch, where W < -1 for x < 0.

err contains an estimate of the absolute error in the value *y*.

This function is from the GNU Scientific Library, see http://www.gnu.org/software/gsl/ for documentation.

Ølog˙1plusx -*- texinfo -*-

`y = log_1plusx (x)` [Loadable Function]
`[y, err] = log_1plusx (...)` [Loadable Function]

These routines compute log(1 + x) for x > -1 using an algorithm that is accurate for small x.

err contains an estimate of the absolute error in the value *y*.

This function is from the GNU Scientific Library, see http://www.gnu.org/software/gsl/ for documentation.

Ølog˙1plusx˙mx -*- texinfo -*-

`y = log_1plusx_mx (x)` [Loadable Function]
`[y, err] = log_1plusx_mx (...)` [Loadable Function]

These routines compute log(1 + x) - x for x > -1 using an algorithm that is accurate for small x.

err contains an estimate of the absolute error in the value *y*.

This function is from the GNU Scientific Library, see http://www.gnu.org/software/gsl/ for documentation.

Øpsi -*- texinfo -*-

y = psi (*x*) [Loadable Function]
[*y*, *err*] = psi (...) [Loadable Function]

These routines compute the digamma function psi(x) for general x, x e 0.

err contains an estimate of the absolute error in the value *y*.

This function is from the GNU Scientific Library, see http://www.gnu.org/software/gsl/ for documentation.

Øpsi˙1piy -*- texinfo -*-

y = psi_1piy (*x*) [Loadable Function]
[*y*, *err*] = psi_1piy (...) [Loadable Function]

These routines compute the real part of the digamma function on the line 1+i y, Re[psi(1 + i y)].

err contains an estimate of the absolute error in the value *y*.

This function is from the GNU Scientific Library, see http://www.gnu.org/software/gsl/ for documentation.

Øsynchrotron˙1 -*- texinfo -*-

y = synchrotron_1 (*x*) [Loadable Function]
[*y*, *err*] = synchrotron_1 (...) [Loadable Function]

These routines compute the first synchrotron function x int˙x^infty dt K˙{5/3}(t) for x >= 0.

err contains an estimate of the absolute error in the value *y*.

This function is from the GNU Scientific Library, see http://www.gnu.org/software/gsl/ for documentation.

Øsynchrotron˙2 -*- texinfo -*-

y = synchrotron_2 (*x*) [Loadable Function]
[*y*, *err*] = synchrotron_2 (...) [Loadable Function]

These routines compute the second synchrotron function x K˙{2/3}(x) for x >= 0.

err contains an estimate of the absolute error in the value *y*.

This function is from the GNU Scientific Library, see http://www.gnu.org/software/gsl/ for documentation.

Øtransport˙2 -*- texinfo -*-

y = transport_2 (*x*) [Loadable Function]
[*y*, *err*] = transport_2 (...) [Loadable Function]

These routines compute the transport function J(2,x).

The transport functions J(n,x) are defined by the integral representations J(n,x) := int˙0^x dt t^n e^t /(e^t - 1)^2.

err contains an estimate of the absolute error in the value *y*.

This function is from the GNU Scientific Library, see http://www.gnu.org/software/gsl/ for documentation.

Øtransport˙3 -*- texinfo -*-

`y = transport_3 (x)` [Loadable Function]
`[y, err] = transport_3 (...)` [Loadable Function]

These routines compute the transport function J(3,x).

The transport functions J(n,x) are defined by the integral representations J(n,x) := int˙0^x dt t^n e^t /(e^t - 1)^2.

err contains an estimate of the absolute error in the value *y*.

This function is from the GNU Scientific Library, see http://www.gnu.org/software/gsl/ for documentation.

Øtransport˙4 -*- texinfo -*-

`y = transport_4 (x)` [Loadable Function]
`[y, err] = transport_4 (...)` [Loadable Function]

These routines compute the transport function J(4,x).

The transport functions J(n,x) are defined by the integral representations J(n,x) := int˙0^x dt t^n e^t /(e^t - 1)^2.

err contains an estimate of the absolute error in the value *y*.

This function is from the GNU Scientific Library, see http://www.gnu.org/software/gsl/ for documentation.

Øtransport˙5 -*- texinfo -*-

`y = transport_5 (x)` [Loadable Function]
`[y, err] = transport_5 (...)` [Loadable Function]

These routines compute the transport function J(5,x).

The transport functions J(n,x) are defined by the integral representations J(n,x) := int˙0^x dt t^n e^t /(e^t - 1)^2.

err contains an estimate of the absolute error in the value *y*.

This function is from the GNU Scientific Library, see http://www.gnu.org/software/gsl/ for documentation.

Øsinc˙gsl -*- texinfo -*-

`y = sinc_gsl (x)` [Loadable Function]
`[y, err] = sinc_gsl (...)` [Loadable Function]

These routines compute sinc(x) = sin(pi x) / (pi x) for any value of x.

err contains an estimate of the absolute error in the value *y*.

This function is from the GNU Scientific Library, see http://www.gnu.org/software/gsl/ for documentation.

Ølnsinh -*- texinfo -*-

`y = lnsinh (x)` [Loadable Function]
`[y, err] = lnsinh (...)` [Loadable Function]

These routines compute log(sinh(x)) for x > 0.

err contains an estimate of the absolute error in the value *y*.

This function is from the GNU Scientific Library, see http://www.gnu.org/software/gsl/ for documentation.

Ølncosh -*- texinfo -*-

`y = lncosh (x)` [Loadable Function]
`[y, err] = lncosh (...)` [Loadable Function]

These routines compute log(cosh(x)) for any x.

err contains an estimate of the absolute error in the value *y*.

This function is from the GNU Scientific Library, see http://www.gnu.org/software/gsl/ for documentation.

Øzeta -*- texinfo -*-

`y = zeta (x)` [Loadable Function]
`[y, err] = zeta (...)` [Loadable Function]

These routines compute the Riemann zeta function zeta(s) for arbitrary s, s e 1.

The Riemann zeta function is defined by the infinite sum zeta(s) = sum˙{k=1}^infty k^{-s}.

err contains an estimate of the absolute error in the value *y*.

This function is from the GNU Scientific Library, see http://www.gnu.org/software/gsl/ for documentation.

Øeta -*- texinfo -*-

`y = eta (x)` [Loadable Function]
`[y, err] = eta (...)` [Loadable Function]

These routines compute the eta function eta(s) for arbitrary s.

The eta function is defined by eta(s) = (1-2^{1-s}) zeta(s).

err contains an estimate of the absolute error in the value *y*.

This function is from the GNU Scientific Library, see http://www.gnu.org/software/gsl/ for documentation.

Øbessel˙Jn -*- texinfo -*-

`y = bessel_Jn (n, x)` [Loadable Function]
`[y, err] = bessel_Jn (...)` [Loadable Function]

These routines compute the regular cylindrical Bessel function of order n, J˙n(x).

err contains an estimate of the absolute error in the value *y*.

This function is from the GNU Scientific Library, see http://www.gnu.org/software/gsl/ for documentation.

Øbessel˙Yn -*- texinfo -*-

`y = bessel_Yn (n, x)` [Loadable Function]
`[y, err] = bessel_Yn (...)` [Loadable Function]

These routines compute the irregular cylindrical Bessel function of order n, Y˙n(x), for x>0.

err contains an estimate of the absolute error in the value *y*.

This function is from the GNU Scientific Library, see http://www.gnu.org/software/gsl/ for documentation.

Øbessel˙In -*- texinfo -*-

y = bessel_In (*n*, *x*) [Loadable Function]
[*y*, *err*] = bessel_In (. . .) [Loadable Function]

These routines compute the regular modified cylindrical Bessel function of order n, I˙n(x).

err contains an estimate of the absolute error in the value *y*.

This function is from the GNU Scientific Library, see http://www.gnu.org/software/gsl/ for documentation.

Øbessel˙In˙scaled -*- texinfo -*-

y = bessel_In_scaled (*n*, *x*) [Loadable Function]
[*y*, *err*] = bessel_In_scaled (. . .) [Loadable Function]

These routines compute the scaled regular modified cylindrical Bessel function of order n, exp(-|x|) I˙n(x)

err contains an estimate of the absolute error in the value *y*.

This function is from the GNU Scientific Library, see http://www.gnu.org/software/gsl/ for documentation.

Øbessel˙Kn -*- texinfo -*-

y = bessel_Kn (*n*, *x*) [Loadable Function]
[*y*, *err*] = bessel_Kn (. . .) [Loadable Function]

These routines compute the irregular modified cylindrical Bessel function of order n, K˙n(x), for x > 0.

err contains an estimate of the absolute error in the value *y*.

This function is from the GNU Scientific Library, see http://www.gnu.org/software/gsl/ for documentation.

Øbessel˙Kn˙scaled -*- texinfo -*-

y = bessel_Kn_scaled (*n*, *x*) [Loadable Function]
[*y*, *err*] = bessel_Kn_scaled (. . .) [Loadable Function]

err contains an estimate of the absolute error in the value *y*.

This function is from the GNU Scientific Library, see http://www.gnu.org/software/gsl/ for documentation.

Øbessel˙jl -*- texinfo -*-

y = bessel_jl (*n*, *x*) [Loadable Function]
[*y*, *err*] = bessel_jl (. . .) [Loadable Function]

These routines compute the regular spherical Bessel function of order l, j˙l(x), for l >= 0 and x >= 0.

err contains an estimate of the absolute error in the value *y*.

This function is from the GNU Scientific Library, see http://www.gnu.org/software/gsl/ for documentation.

Øbessel˙yl -*- texinfo -*-

`y = bessel_yl (n, x)` [Loadable Function]
`[y, err] = bessel_yl (...)` [Loadable Function]

These routines compute the irregular spherical Bessel function of order l, y˙l(x), for l >= 0.

err contains an estimate of the absolute error in the value *y*.

This function is from the GNU Scientific Library, see http://www.gnu.org/software/gsl/ for documentation.

Øbessel˙il˙scaled -*- texinfo -*-

`y = bessel_il_scaled (n, x)` [Loadable Function]
`[y, err] = bessel_il_scaled (...)` [Loadable Function]

These routines compute the scaled regular modified spherical Bessel function of order l, exp(-|x|) i˙l(x)

err contains an estimate of the absolute error in the value *y*.

This function is from the GNU Scientific Library, see http://www.gnu.org/software/gsl/ for documentation.

Øbessel˙kl˙scaled -*- texinfo -*-

`y = bessel_kl_scaled (n, x)` [Loadable Function]
`[y, err] = bessel_kl_scaled (...)` [Loadable Function]

These routines compute the scaled irregular modified spherical Bessel function of order l, exp(x) k˙l(x), for x>0.

err contains an estimate of the absolute error in the value *y*.

This function is from the GNU Scientific Library, see http://www.gnu.org/software/gsl/ for documentation.

Øexprel˙n -*- texinfo -*-

`y = exprel_n (n, x)` [Loadable Function]
`[y, err] = exprel_n (...)` [Loadable Function]

These routines compute the N-relative exponential, which is the n-th generalization of the functions gsl˙sf˙exprel and gsl˙sf˙exprel2. The N-relative exponential is given by,

exprel˙N(x) = N!/x^N (exp(x) - sum˙{k=0}^{N-1} x^k/k!) = 1 + x/(N+1) + x^2/((N+1)(N+2)) + ... = 1F1 (1,1+N,x)

err contains an estimate of the absolute error in the value *y*.

This function is from the GNU Scientific Library, see http://www.gnu.org/software/gsl/ for documentation.

Øfermi˙dirac˙int -*- texinfo -*-

`y = fermi_dirac_int (n, x)` [Loadable Function]
`[y, err] = fermi_dirac_int (...)` [Loadable Function]

These routines compute the complete Fermi-Dirac integral with an integer index of j, F˙j(x) = (1/Gamma(j+1)) int˙0^infty dt (t^j /(exp(t-x)+1)).

err contains an estimate of the absolute error in the value *y*.

This function is from the GNU Scientific Library, see http://www.gnu.org/software/gsl/ for documentation.

Øtaylorcoeff -*- texinfo -*-

`y = taylorcoeff (n, x)` [Loadable Function]
`[y, err] = taylorcoeff (...)` [Loadable Function]

These routines compute the Taylor coefficient x^n / n! for x >= 0, n >= 0.

err contains an estimate of the absolute error in the value *y*.

This function is from the GNU Scientific Library, see http://www.gnu.org/software/gsl/ for documentation.

Ølegendre˙Pl -*- texinfo -*-

`y = legendre_Pl (n, x)` [Loadable Function]
`[y, err] = legendre_Pl (...)` [Loadable Function]

These functions evaluate the Legendre polynomial P˙l(x) for a specific value of l, x subject to l >= 0, |x| <= 1

err contains an estimate of the absolute error in the value *y*.

This function is from the GNU Scientific Library, see http://www.gnu.org/software/gsl/ for documentation.

Ølegendre˙Ql -*- texinfo -*-

`y = legendre_Ql (n, x)` [Loadable Function]
`[y, err] = legendre_Ql (...)` [Loadable Function]

These routines compute the Legendre function Q˙l(x) for x > -1, x != 1 and l >= 0.

err contains an estimate of the absolute error in the value *y*.

This function is from the GNU Scientific Library, see http://www.gnu.org/software/gsl/ for documentation.

Øpsi˙n -*- texinfo -*-

`y = psi_n (n, x)` [Loadable Function]
`[y, err] = psi_n (...)` [Loadable Function]

These routines compute the polygamma function psi^{(m)}(x) for m >= 0, x > 0.

err contains an estimate of the absolute error in the value *y*.

This function is from the GNU Scientific Library, see http://www.gnu.org/software/gsl/ for documentation.

Øbessel˙Jnu -*- texinfo -*-

`z = bessel_Jnu (x, y)` [Loadable Function]
`[z, err] = bessel_Jnu (...)` [Loadable Function]

These routines compute the regular cylindrical Bessel function of fractional order nu, J˙u(x).

err contains an estimate of the absolute error in the value *z*.

This function is from the GNU Scientific Library, see http://www.gnu.org/software/gsl/ for documentation.

Øbessel˙Ynu -*- texinfo -*-

`z = bessel_Ynu (x, y)` [Loadable Function]
`[z, err] = bessel_Ynu (...)` [Loadable Function]

These routines compute the irregular cylindrical Bessel function of fractional order nu, Y˙u(x).

err contains an estimate of the absolute error in the value *z*.

This function is from the GNU Scientific Library, see http://www.gnu.org/software/gsl/ for documentation.

Øbessel˙Inu -*- texinfo -*-

`z = bessel_Inu (x, y)` [Loadable Function]
`[z, err] = bessel_Inu (...)` [Loadable Function]

These routines compute the regular modified Bessel function of fractional order nu, I˙u(x) for x>0, u>0.

err contains an estimate of the absolute error in the value *z*.

This function is from the GNU Scientific Library, see http://www.gnu.org/software/gsl/ for documentation.

Øbessel˙Inu˙scaled -*- texinfo -*-

`z = bessel_Inu_scaled (x, y)` [Loadable Function]
`[z, err] = bessel_Inu_scaled (...)` [Loadable Function]

These routines compute the scaled regular modified Bessel function of fractional order nu, exp(-|x|)I˙u(x) for x>0, u>0.

err contains an estimate of the absolute error in the value *z*.

This function is from the GNU Scientific Library, see http://www.gnu.org/software/gsl/ for documentation.

Øbessel˙Knu -*- texinfo -*-

`z = bessel_Knu (x, y)` [Loadable Function]
`[z, err] = bessel_Knu (...)` [Loadable Function]

These routines compute the irregular modified Bessel function of fractional order nu, K˙u(x) for x>0, u>0.

err contains an estimate of the absolute error in the value *z*.

This function is from the GNU Scientific Library, see http://www.gnu.org/software/gsl/ for documentation.

Øbessel˙lnKnu -*- texinfo -*-

`z = bessel_lnKnu (x, y)` [Loadable Function]
`[z, err] = bessel_lnKnu (...)` [Loadable Function]

These routines compute the logarithm of the irregular modified Bessel function of fractional order nu, ln(K˙u(x)) for x>0, u>0.

err contains an estimate of the absolute error in the value *z*.

This function is from the GNU Scientific Library, see http://www.gnu.org/software/gsl/ for documentation.

Øbessel˙Knu˙scaled -*- texinfo -*-

`z = bessel_Knu_scaled (x, y)` [Loadable Function]
`[z, err] = bessel_Knu_scaled (...)` [Loadable Function]

These routines compute the scaled irregular modified Bessel function of fractional order nu, exp(+|x|) K˙u(x) for x>0, u>0.

err contains an estimate of the absolute error in the value *z*.

This function is from the GNU Scientific Library, see http://www.gnu.org/software/gsl/ for documentation.

Øexp˙mult -*- texinfo -*-

`z = exp_mult (x, y)` [Loadable Function]
`[z, err] = exp_mult (...)` [Loadable Function]

These routines exponentiate x and multiply by the factor y to return the product y exp(x).

err contains an estimate of the absolute error in the value *z*.

This function is from the GNU Scientific Library, see http://www.gnu.org/software/gsl/ for documentation.

Øfermi˙dirac˙inc˙0 -*- texinfo -*-

`z = fermi_dirac_inc_0 (x, y)` [Loadable Function]
`[z, err] = fermi_dirac_inc_0 (...)` [Loadable Function]

These routines compute the incomplete Fermi-Dirac integral with an index of zero, F˙0(x,b) = ln(1 + e^{b-x}) - (b-x).

err contains an estimate of the absolute error in the value *z*.

This function is from the GNU Scientific Library, see http://www.gnu.org/software/gsl/ for documentation.

Øpoch -*- texinfo -*-

`z = poch (x, y)` [Loadable Function]
`[z, err] = poch (...)` [Loadable Function]

These routines compute the Pochhammer symbol

(a)˙x := Gamma(a + x)/Gamma(a),

subject to a and a+x not being negative integers. The Pochhammer symbol is also known as the Apell symbol.

err contains an estimate of the absolute error in the value *z*.

This function is from the GNU Scientific Library, see http://www.gnu.org/software/gsl/ for documentation.

Ølnpoch -*- texinfo -*-

`z = lnpoch (x, y)` [Loadable Function]
`[z, err] = lnpoch (...)` [Loadable Function]

These routines compute the logarithm of the Pochhammer symbol, log((a)˙x) = log(Gamma(a + x)/Gamma(a)) for a > 0, a+x > 0.

err contains an estimate of the absolute error in the value *z*.

This function is from the GNU Scientific Library, see http://www.gnu.org/software/gsl/ for documentation.

Øpochrel -*- texinfo -*-

`z = pochrel (x, y)` [Loadable Function]
`[z, err] = pochrel (...)` [Loadable Function]

These routines compute the relative Pochhammer symbol ((a,x) - 1)/x where (a,x) = (a)˙x := Gamma(a + x)/Gamma(a).

err contains an estimate of the absolute error in the value *z*.

This function is from the GNU Scientific Library, see http://www.gnu.org/software/gsl/ for documentation.

Øgamma˙inc˙Q -*- texinfo -*-

`z = gamma_inc_Q (x, y)` [Loadable Function]
`[z, err] = gamma_inc_Q (...)` [Loadable Function]

These routines compute the normalized incomplete Gamma Function Q(a,x) = 1/Gamma(a) int˙xinfty dt t^{a-1} exp(-t) for a > 0, x >= 0.

err contains an estimate of the absolute error in the value *z*.

This function is from the GNU Scientific Library, see http://www.gnu.org/software/gsl/ for documentation.

Øgamma˙inc˙P -*- texinfo -*-

`z = gamma_inc_P (x, y)` [Loadable Function]
`[z, err] = gamma_inc_P (...)` [Loadable Function]

These routines compute the complementary normalized incomplete Gamma Function P(a,x) = 1/Gamma(a) int˙0^x dt t^{a-1} exp(-t) for a > 0, x >= 0.

err contains an estimate of the absolute error in the value *z*.

This function is from the GNU Scientific Library, see http://www.gnu.org/software/gsl/ for documentation.

Øgamma˙inc -*- texinfo -*-

`z = gamma_inc` (*x*, *y*) [Loadable Function]
`[z, err] = gamma_inc` (. . .) [Loadable Function]

These functions compute the incomplete Gamma Function the normalization factor included in the previously defined functions: Gamma(a,x) = int˙xinfty dt t^{a-1} exp(-t) for a real and x >= 0.

err contains an estimate of the absolute error in the value *z*.

This function is from the GNU Scientific Library, see `http://www.gnu.org/software/gsl/` for documentation.

Øbeta˙gsl -*- texinfo -*-

`z = beta_gsl` (*x*, *y*) [Loadable Function]
`[z, err] = beta_gsl` (. . .) [Loadable Function]

These routines compute the Beta Function, B(a,b) = Gamma(a)Gamma(b)/Gamma(a+b) for a > 0, b > 0.

err contains an estimate of the absolute error in the value *z*.

This function is from the GNU Scientific Library, see `http://www.gnu.org/software/gsl/` for documentation.

Ølnbeta -*- texinfo -*-

`z = lnbeta` (*x*, *y*) [Loadable Function]
`[z, err] = lnbeta` (. . .) [Loadable Function]

These routines compute the logarithm of the Beta Function, log(B(a,b)) for a > 0, b > 0.

err contains an estimate of the absolute error in the value *z*.

This function is from the GNU Scientific Library, see `http://www.gnu.org/software/gsl/` for documentation.

Øhyperg˙0F1 -*- texinfo -*-

`z = hyperg_0F1` (*x*, *y*) [Loadable Function]
`[z, err] = hyperg_0F1` (. . .) [Loadable Function]

These routines compute the hypergeometric function 0F1(c,x).

err contains an estimate of the absolute error in the value *z*.

This function is from the GNU Scientific Library, see `http://www.gnu.org/software/gsl/` for documentation.

ØconicalP˙half -*- texinfo -*-

`z = conicalP_half` (*x*, *y*) [Loadable Function]
`[z, err] = conicalP_half` (. . .) [Loadable Function]

These routines compute the irregular Spherical Conical Function P^{1/2}˙{-1/2 + i lambda}(x) for x > -1.

err contains an estimate of the absolute error in the value *z*.

This function is from the GNU Scientific Library, see `http://www.gnu.org/software/gsl/` for documentation.

ØconicalP˙mhalf -*- texinfo -*-

`z = conicalP_mhalf (x, y)` [Loadable Function]
`[z, err] = conicalP_mhalf (...)` [Loadable Function]

These routines compute the regular Spherical Conical Function P^{-1/2}˙{-1/2 + i lambda}(x) for x > -1.

err contains an estimate of the absolute error in the value *z*.

This function is from the GNU Scientific Library, see http://www.gnu.org/software/gsl/ for documentation.

ØconicalP˙0 -*- texinfo -*-

`z = conicalP_0 (x, y)` [Loadable Function]
`[z, err] = conicalP_0 (...)` [Loadable Function]

These routines compute the conical function P^0˙{-1/2 + i lambda}(x) for x > -1.

err contains an estimate of the absolute error in the value *z*.

This function is from the GNU Scientific Library, see http://www.gnu.org/software/gsl/ for documentation.

ØconicalP˙1 -*- texinfo -*-

`z = conicalP_1 (x, y)` [Loadable Function]
`[z, err] = conicalP_1 (...)` [Loadable Function]

These routines compute the conical function P^1˙{-1/2 + i lambda}(x) for x > -1.

err contains an estimate of the absolute error in the value *z*.

This function is from the GNU Scientific Library, see http://www.gnu.org/software/gsl/ for documentation.

Øhzeta -*- texinfo -*-

`z = hzeta (x, y)` [Loadable Function]
`[z, err] = hzeta (...)` [Loadable Function]

These routines compute the Hurwitz zeta function zeta(s,q) for s > 1, q > 0.

err contains an estimate of the absolute error in the value *z*.

This function is from the GNU Scientific Library, see http://www.gnu.org/software/gsl/ for documentation.

Øairy˙Ai -*- texinfo -*-

`y = airy_Ai (x, mode)` [Loadable Function]
`[y, err] = airy_Ai (...)` [Loadable Function]

These routines compute the Airy function Ai(x) with an accuracy specified by mode.

The second argument *mode* must be an integer corresponding to

0 = GSL˙PREC˙DOUBLE
: Double-precision, a relative accuracy of approximately `2 * 10^-16`.

1 = GSL˙PREC˙SINGLE
: Single-precision, a relative accuracy of approximately `10^-7`.

2 = GSL˙PREC˙APPROX
Approximate values, a relative accuracy of approximately `5 * 10^-4`.

err contains an estimate of the absolute error in the value *y*.

This function is from the GNU Scientific Library, see http://www.gnu.org/software/gsl/ for documentation.

Øairy˙Bi -*- texinfo -*-

`y = airy_Bi (x, mode)` [Loadable Function]
`[y, err] = airy_Bi (...)` [Loadable Function]

These routines compute the Airy function Bi(x) with an accuracy specified by mode.

The second argument *mode* must be an integer corresponding to

0 = GSL˙PREC˙DOUBLE
Double-precision, a relative accuracy of approximately `2 * 10^-16`.

1 = GSL˙PREC˙SINGLE
Single-precision, a relative accuracy of approximately `10^-7`.

2 = GSL˙PREC˙APPROX
Approximate values, a relative accuracy of approximately `5 * 10^-4`.

err contains an estimate of the absolute error in the value *y*.

This function is from the GNU Scientific Library, see http://www.gnu.org/software/gsl/ for documentation.

Øairy˙Ai˙scaled -*- texinfo -*-

`y = airy_Ai_scaled (x, mode)` [Loadable Function]
`[y, err] = airy_Ai_scaled (...)` [Loadable Function]

These routines compute a scaled version of the Airy function S˙A(x) Ai(x). For x>0 the scaling factor S˙A(x) is exp(+(2/3) x^(3/2)), and is 1 for x<0.

The second argument *mode* must be an integer corresponding to

0 = GSL˙PREC˙DOUBLE
Double-precision, a relative accuracy of approximately `2 * 10^-16`.

1 = GSL˙PREC˙SINGLE
Single-precision, a relative accuracy of approximately `10^-7`.

2 = GSL˙PREC˙APPROX
Approximate values, a relative accuracy of approximately `5 * 10^-4`.

err contains an estimate of the absolute error in the value *y*.

This function is from the GNU Scientific Library, see http://www.gnu.org/software/gsl/ for documentation.

Øairy˙Bi˙scaled -*- texinfo -*-

`y = airy_Bi_scaled (x, mode)` [Loadable Function]
`[y, err] = airy_Bi_scaled (...)` [Loadable Function]

These routines compute a scaled version of the Airy function S˙B(x) Bi(x). For x>0 the scaling factor S˙B(x) is exp(-(2/3) x^(3/2)), and is 1 for x<0.

The second argument *mode* must be an integer corresponding to

0 = GSL˙PREC˙DOUBLE
: Double-precision, a relative accuracy of approximately `2 * 10^-16`.

1 = GSL˙PREC˙SINGLE
: Single-precision, a relative accuracy of approximately `10^-7`.

2 = GSL˙PREC˙APPROX
: Approximate values, a relative accuracy of approximately `5 * 10^-4`.

err contains an estimate of the absolute error in the value *y*.

This function is from the GNU Scientific Library, see http://www.gnu.org/software/gsl/ for documentation.

Øairy˙Ai˙deriv -*- texinfo -*-

`y = airy_Ai_deriv (x, mode)` [Loadable Function]
`[y, err] = airy_Ai_deriv (...)` [Loadable Function]

These routines compute the Airy function derivative Ai'(x) with an accuracy specified by mode.

The second argument *mode* must be an integer corresponding to

0 = GSL˙PREC˙DOUBLE
: Double-precision, a relative accuracy of approximately `2 * 10^-16`.

1 = GSL˙PREC˙SINGLE
: Single-precision, a relative accuracy of approximately `10^-7`.

2 = GSL˙PREC˙APPROX
: Approximate values, a relative accuracy of approximately `5 * 10^-4`.

err contains an estimate of the absolute error in the value *y*.

This function is from the GNU Scientific Library, see http://www.gnu.org/software/gsl/ for documentation.

Øairy˙Bi˙deriv -*- texinfo -*-

`y = airy_Bi_deriv (x, mode)` [Loadable Function]
`[y, err] = airy_Bi_deriv (...)` [Loadable Function]

These routines compute the Airy function derivative Bi'(x) with an accuracy specified by mode.

The second argument *mode* must be an integer corresponding to

0 = GSL˙PREC˙DOUBLE
: Double-precision, a relative accuracy of approximately `2 * 10^-16`.

1 = GSL˙PREC˙SINGLE
: Single-precision, a relative accuracy of approximately `10^-7`.

2 = GSL˙PREC˙APPROX
Approximate values, a relative accuracy of approximately `5 * 10^-4`.

err contains an estimate of the absolute error in the value *y*.

This function is from the GNU Scientific Library, see http://www.gnu.org/software/gsl/ for documentation.

Øairy˙Ai˙deriv˙scaled -*- texinfo -*-

y = airy_Ai_deriv_scaled (*x*, *mode*) [Loadable Function]
[*y*, *err*] = airy_Ai_deriv_scaled (...) [Loadable Function]

These routines compute the derivative of the scaled Airy function S˙A(x) Ai(x).

The second argument *mode* must be an integer corresponding to

0 = GSL˙PREC˙DOUBLE
Double-precision, a relative accuracy of approximately `2 * 10^-16`.

1 = GSL˙PREC˙SINGLE
Single-precision, a relative accuracy of approximately `10^-7`.

2 = GSL˙PREC˙APPROX
Approximate values, a relative accuracy of approximately `5 * 10^-4`.

err contains an estimate of the absolute error in the value *y*.

This function is from the GNU Scientific Library, see http://www.gnu.org/software/gsl/ for documentation.

Øairy˙Bi˙deriv˙scaled -*- texinfo -*-

y = airy_Bi_deriv_scaled (*x*, *mode*) [Loadable Function]
[*y*, *err*] = airy_Bi_deriv_scaled (...) [Loadable Function]

These routines compute the derivative of the scaled Airy function S˙B(x) Bi(x).

The second argument *mode* must be an integer corresponding to

0 = GSL˙PREC˙DOUBLE
Double-precision, a relative accuracy of approximately `2 * 10^-16`.

1 = GSL˙PREC˙SINGLE
Single-precision, a relative accuracy of approximately `10^-7`.

2 = GSL˙PREC˙APPROX
Approximate values, a relative accuracy of approximately `5 * 10^-4`.

err contains an estimate of the absolute error in the value *y*.

This function is from the GNU Scientific Library, see http://www.gnu.org/software/gsl/ for documentation.

Øellint˙Kcomp -*- texinfo -*-

y = ellint_Kcomp (*x*, *mode*) [Loadable Function]

[y, err] = ellint_Kcomp (...) [Loadable Function]

These routines compute the complete elliptic integral K(k)

$$K(k) = \int_0^{\pi/2} \frac{dt}{\sqrt{(1 - k^2 \sin^2(t))}}$$

See also:

ellipj, ellipke

The notation used here is based on Carlson, *Numerische Mathematik* 33 (1979) and differs slightly from that used by Abramowitz & Stegun, where the functions are given in terms of the parameter $m = k^2$.

The second argument *mode* must be an integer corresponding to

0 = GSL˙PREC˙DOUBLE
: Double-precision, a relative accuracy of approximately `2 * 10^-16`.

1 = GSL˙PREC˙SINGLE
: Single-precision, a relative accuracy of approximately `10^-7`.

2 = GSL˙PREC˙APPROX
: Approximate values, a relative accuracy of approximately `5 * 10^-4`.

err contains an estimate of the absolute error in the value *y*.

This function is from the GNU Scientific Library, see http://www.gnu.org/software/gsl/ for documentation.

Øellint˙Ecomp -*- texinfo -*-

y = ellint_Ecomp (x, mode) [Loadable Function]

[y, err] = ellint_Ecomp (...) [Loadable Function]

These routines compute the complete elliptic integral E(k) to the accuracy specified by the mode variable mode.

$$E(k) = \int_0^{\pi/2} \sqrt{(1 - k^2 \sin^2(t))} dt$$

See also:

ellipj, ellipke

The notation used here is based on Carlson, *Numerische Mathematik* 33 (1979) and differs slightly from that used by Abramowitz & Stegun, where the functions are given in terms of the parameter $m = k^2$.

The second argument *mode* must be an integer corresponding to

0 = GSL˙PREC˙DOUBLE
: Double-precision, a relative accuracy of approximately `2 * 10^-16`.

1 = GSL˙PREC˙SINGLE
: Single-precision, a relative accuracy of approximately `10^-7`.

2 = GSL˙PREC˙APPROX
: Approximate values, a relative accuracy of approximately `5 * 10^-4`.

err contains an estimate of the absolute error in the value *y*.

This function is from the GNU Scientific Library, see http://www.gnu.org/software/gsl/ for documentation.

Øairy˙zero˙Ai -*- texinfo -*-

`y = airy_zero_Ai (n)` [Loadable Function]
`[y, err] = airy_zero_Ai (...)` [Loadable Function]

These routines compute the location of the s-th zero of the Airy function Ai(x).

err contains an estimate of the absolute error in the value *y*.

This function is from the GNU Scientific Library, see http://www.gnu.org/software/gsl/ for documentation.

Øairy˙zero˙Bi -*- texinfo -*-

`y = airy_zero_Bi (n)` [Loadable Function]
`[y, err] = airy_zero_Bi (...)` [Loadable Function]

These routines compute the location of the s-th zero of the Airy function Bi(x).

err contains an estimate of the absolute error in the value *y*.

This function is from the GNU Scientific Library, see http://www.gnu.org/software/gsl/ for documentation.

Øairy˙zero˙Ai˙deriv -*- texinfo -*-

`y = airy_zero_Ai_deriv (n)` [Loadable Function]
`[y, err] = airy_zero_Ai_deriv (...)` [Loadable Function]

These routines compute the location of the s-th zero of the Airy function derivative Ai(x).

err contains an estimate of the absolute error in the value *y*.

This function is from the GNU Scientific Library, see http://www.gnu.org/software/gsl/ for documentation.

Øairy˙zero˙Bi˙deriv -*- texinfo -*-

`y = airy_zero_Bi_deriv (n)` [Loadable Function]
`[y, err] = airy_zero_Bi_deriv (...)` [Loadable Function]

These routines compute the location of the s-th zero of the Airy function derivative Bi(x).

err contains an estimate of the absolute error in the value *y*.

This function is from the GNU Scientific Library, see http://www.gnu.org/software/gsl/ for documentation.

Øbessel˙zero˙J0 -*- texinfo -*-

`y = bessel_zero_J0 (n)` [Loadable Function]
`[y, err] = bessel_zero_J0 (...)` [Loadable Function]

These routines compute the location of the s-th positive zero of the Bessel function J˙0(x).

err contains an estimate of the absolute error in the value *y*.

This function is from the GNU Scientific Library, see http://www.gnu.org/software/gsl/ for documentation.

Øbessel˙zero˙J1 -*- texinfo -*-

`y = bessel_zero_J1 (n)` [Loadable Function]
`[y, err] = bessel_zero_J1 (...)` [Loadable Function]

These routines compute the location of the s-th positive zero of the Bessel function J˙1(x).

err contains an estimate of the absolute error in the value *y*.

This function is from the GNU Scientific Library, see http://www.gnu.org/software/gsl/ for documentation.

Øpsi˙1˙int -*- texinfo -*-

`y = psi_1_int (n)` [Loadable Function]
`[y, err] = psi_1_int (...)` [Loadable Function]

These routines compute the Trigamma function psi(n) for positive integer n.

err contains an estimate of the absolute error in the value *y*.

This function is from the GNU Scientific Library, see http://www.gnu.org/software/gsl/ for documentation.

Øzeta˙int -*- texinfo -*-

`y = zeta_int (n)` [Loadable Function]
`[y, err] = zeta_int (...)` [Loadable Function]

These routines compute the Riemann zeta function zeta(n) for integer n, n e 1.

err contains an estimate of the absolute error in the value *y*.

This function is from the GNU Scientific Library, see http://www.gnu.org/software/gsl/ for documentation.

Øeta˙int -*- texinfo -*-

`y = eta_int (n)` [Loadable Function]
`[y, err] = eta_int (...)` [Loadable Function]

These routines compute the eta function eta(n) for integer n.

err contains an estimate of the absolute error in the value *y*.

This function is from the GNU Scientific Library, see http://www.gnu.org/software/gsl/ for documentation.

Ølegendre˙Plm -*- texinfo -*-

`y = legendre_Plm (n, m, x)` [Loadable Function]
`[y, err] = legendre_Plm (...)` [Loadable Function]

These routines compute the associated Legendre polynomial P˙l^m(x) for m >= 0, l >= m, |x| <= 1.

err contains an estimate of the absolute error in the value *y*.

This function is from the GNU Scientific Library, see http://www.gnu.org/software/gsl/ for documentation.

Ølegendre˙sphPlm -*- texinfo -*-

`y = legendre_sphPlm (n, m, x)` [Loadable Function]
`[y, err] = legendre_sphPlm (...)` [Loadable Function]

These routines compute the normalized associated Legendre polynomial $sqrt{(2l+1)/(4pi)} sqrt{(l-m)!/(l+m)!} P˙l^m(x)$ suitable for use in spherical harmonics. The parameters must satisfy m >= 0, l >= m, |x| <= 1. Theses routines avoid the overflows that occur for the standard normalization of P˙l^m(x).

err contains an estimate of the absolute error in the value *y*.

This function is from the GNU Scientific Library, see http://www.gnu.org/software/gsl/ for documentation.

Øhyperg˙U -*- texinfo -*-

`out = hyperg_U (x0, x1, x2)` [Loadable Function]
`[out, err] = hyperg_U (...)` [Loadable Function]

Secondary Confluent Hypergoemetric U function A&E 13.1.3 All inputs are double as is the output.

err contains an estimate of the absolute error in the value *out*.a.

This function is from the GNU Scientific Library, see http://www.gnu.org/software/gsl/ for documentation.

Øhyperg˙1F1 -*- texinfo -*-

`out = hyperg_1F1 (x0, x1, x2)` [Loadable Function]
`[out, err] = hyperg_1F1 (...)` [Loadable Function]

Primary Confluent Hypergoemetric U function A&E 13.1.3 All inputs are double as is the output.

err contains an estimate of the absolute error in the value *out*.a.

This function is from the GNU Scientific Library, see http://www.gnu.org/software/gsl/ for documentation.

Øgsl˙sf -*- texinfo -*-

`gsl_sf ()` [Loadable Function]

Octave bindings to the GNU Scientific Library. All GSL functions can be called with by the GSL names within octave.

Øclausen -*- texinfo -*-

`y = clausen (x)` [Loadable Function]
`[y, err] = clausen (...)` [Loadable Function]

The Clausen function is defined by the following integral,

Cl˙2(x) = - int˙0^x dt log(2 sin(t/2))

It is related to the dilogarithm by Cl˙2(theta) = Im Li˙2(exp(i theta)).

err contains an estimate of the absolute error in the value *y*.

This function is from the GNU Scientific Library, see http://www.gnu.org/software/gsl/ for documentation.

Ødawson -*- texinfo -*-

`y = dawson (x)` [Loadable Function]
`[y, err] = dawson (...)` [Loadable Function]

The Dawson integral is defined by exp(-x^2) int˙0^x dt exp(t^2). A table of Dawson integral can be found in Abramowitz & Stegun, Table 7.5.

err contains an estimate of the absolute error in the value *y*.

This function is from the GNU Scientific Library, see http://www.gnu.org/software/gsl/ for documentation.

Ødebye˙1 -*- texinfo -*-

`y = debye_1 (x)` [Loadable Function]
`[y, err] = debye_1 (...)` [Loadable Function]

The Debye functions are defined by the integral

D˙n(x) = n/x^n int˙0^x dt (t^n/(e^t - 1)).

For further information see Abramowitz & Stegun, Section 27.1.

err contains an estimate of the absolute error in the value *y*.

This function is from the GNU Scientific Library, see http://www.gnu.org/software/gsl/ for documentation.

Ødebye˙2 -*- texinfo -*-

`y = debye_2 (x)` [Loadable Function]
`[y, err] = debye_2 (...)` [Loadable Function]

The Debye functions are defined by the integral

D˙n(x) = n/x^n int˙0^x dt (t^n/(e^t - 1)).

For further information see Abramowitz & Stegun, Section 27.1.

err contains an estimate of the absolute error in the value *y*.

This function is from the GNU Scientific Library, see http://www.gnu.org/software/gsl/ for documentation.

Ødebye˙3 -*- texinfo -*-

`y = debye_3 (x)` [Loadable Function]
`[y, err] = debye_3 (...)` [Loadable Function]

The Debye functions are defined by the integral

D˙n(x) = n/x^n int˙0^x dt (t^n/(e^t - 1)).

For further information see Abramowitz & Stegun, Section 27.1.

err contains an estimate of the absolute error in the value *y*.

This function is from the GNU Scientific Library, see http://www.gnu.org/software/gsl/ for documentation.

Ødebye˙4 -*- texinfo -*-

y = debye_4 (x) [Loadable Function]
[y, *err*] = debye_4 (...) [Loadable Function]

The Debye functions are defined by the integral

D˙n(x) = n/x^n int˙0^x dt (t^n/(e^t - 1)).

For further information see Abramowitz & Stegun, Section 27.1.

err contains an estimate of the absolute error in the value y.

This function is from the GNU Scientific Library, see http://www.gnu.org/software/gsl/ for documentation.

Øerf˙gsl -*- texinfo -*-

y = erf_gsl (x) [Loadable Function]
[y, *err*] = erf_gsl (...) [Loadable Function]

These routines compute the error function erf(x) = (2/sqrt(pi)) int˙0^x dt exp(-t^2).

err contains an estimate of the absolute error in the value y.

This function is from the GNU Scientific Library, see http://www.gnu.org/software/gsl/ for documentation.

Øerfc˙gsl -*- texinfo -*-

y = erfc_gsl (x) [Loadable Function]
[y, *err*] = erfc_gsl (...) [Loadable Function]

These routines compute the complementary error function erfc(x) = 1 - erf(x) = (2/sqrt(pi)) int˙x^infty exp(-t^2).

err contains an estimate of the absolute error in the value y.

This function is from the GNU Scientific Library, see http://www.gnu.org/software/gsl/ for documentation.

Ølog˙erfc -*- texinfo -*-

y = log_erfc (x) [Loadable Function]
[y, *err*] = log_erfc (...) [Loadable Function]

These routines compute the logarithm of the complementary error function log(erfc(x)).

err contains an estimate of the absolute error in the value y.

This function is from the GNU Scientific Library, see http://www.gnu.org/software/gsl/ for documentation.

Øerf˙Z -*- texinfo -*-

y = erf_Z (x) [Loadable Function]
[y, *err*] = erf_Z (...) [Loadable Function]

These routines compute the Gaussian probability function Z(x) = (1/(2pi)) exp(-x^2/2).

err contains an estimate of the absolute error in the value y.

This function is from the GNU Scientific Library, see http://www.gnu.org/software/gsl/ for documentation.

Øerf˙Q -*- texinfo -*-

y = **erf_Q** (*x*) [Loadable Function]
[*y*, *err*] = **erf_Q** (...) [Loadable Function]

These routines compute the upper tail of the Gaussian probability function Q(x) = (1/(2pi)) int˙x^infty dt exp(-t^2/2).

err contains an estimate of the absolute error in the value *y*.

This function is from the GNU Scientific Library, see http://www.gnu.org/software/gsl/ for documentation.

Øhazard -*- texinfo -*-

y = **hazard** (*x*) [Loadable Function]
[*y*, *err*] = **hazard** (...) [Loadable Function]

The hazard function for the normal distrbution, also known as the inverse Mill's ratio, is defined as h(x) = Z(x)/Q(x) = sqrt{2/pi exp(-x^2 / 2) / erfc(x/sqrt 2)}. It decreases rapidly as x approaches -infty and asymptotes to h(x) sim x as x approaches +infty.

err contains an estimate of the absolute error in the value *y*.

This function is from the GNU Scientific Library, see http://www.gnu.org/software/gsl/ for documentation.

Øexpm1 -*- texinfo -*-

y = **expm1** (*x*) [Loadable Function]
[*y*, *err*] = **expm1** (...) [Loadable Function]

These routines compute the quantity exp(x)-1 using an algorithm that is accurate for small x.

err contains an estimate of the absolute error in the value *y*.

This function is from the GNU Scientific Library, see http://www.gnu.org/software/gsl/ for documentation.

Øexprel -*- texinfo -*-

y = **exprel** (*x*) [Loadable Function]
[*y*, *err*] = **exprel** (...) [Loadable Function]

These routines compute the quantity (exp(x)-1)/x using an algorithm that is accurate for small x. For small x the algorithm is based on the expansion (exp(x)-1)/x = 1 + x/2 + x^2/(2*3) + x^3/(2*3*4) + dots.

err contains an estimate of the absolute error in the value *y*.

This function is from the GNU Scientific Library, see http://www.gnu.org/software/gsl/ for documentation.

Øexprel˙2 -*- texinfo -*-

`y = exprel_2 (x)` [Loadable Function]
`[y, err] = exprel_2 (...)` [Loadable Function]

These routines compute the quantity 2(exp(x)-1-x)/x^2 using an algorithm that is accurate for small x. For small x the algorithm is based on the expansion 2(exp(x)-1-x)/x^2 = 1 + x/3 + x^2/(3*4) + x^3/(3*4*5) + dots.

err contains an estimate of the absolute error in the value *y*.

This function is from the GNU Scientific Library, see http://www.gnu.org/software/gsl/ for documentation.

Øexpint˙E1 -*- texinfo -*-

`y = expint_E1 (x)` [Loadable Function]
`[y, err] = expint_E1 (...)` [Loadable Function]

These routines compute the exponential integral E˙1(x),

E˙1(x) := Re int˙1^infty dt exp(-xt)/t.

err contains an estimate of the absolute error in the value *y*.

This function is from the GNU Scientific Library, see http://www.gnu.org/software/gsl/ for documentation.

Øexpint˙E2 -*- texinfo -*-

`y = expint_E2 (x)` [Loadable Function]
`[y, err] = expint_E2 (...)` [Loadable Function]

These routines compute the second-order exponential integral E˙2(x),

E˙2(x) := Re int˙1^infty dt exp(-xt)/t^2.

err contains an estimate of the absolute error in the value *y*.

This function is from the GNU Scientific Library, see http://www.gnu.org/software/gsl/ for documentation.

Øexpint˙Ei -*- texinfo -*-

`y = expint_Ei (x)` [Loadable Function]
`[y, err] = expint_Ei (...)` [Loadable Function]

These routines compute the exponential integral E˙i(x),

Ei(x) := - PV(int˙{-x}^infty dt exp(-t)/t)

where PV denotes the principal value of the integral.

err contains an estimate of the absolute error in the value *y*.

This function is from the GNU Scientific Library, see http://www.gnu.org/software/gsl/ for documentation.

ØShi -*- texinfo -*-

`y = Shi (x)` [Loadable Function]
`[y, err] = Shi (...)` [Loadable Function]

These routines compute the integral Shi(x) = int˙0^x dt sinh(t)/t.

err contains an estimate of the absolute error in the value *y*.

This function is from the GNU Scientific Library, see http://www.gnu.org/software/gsl/ for documentation.

ØChi -*- texinfo -*-

`y = Chi (x)` [Loadable Function]
`[y, err] = Chi (...)` [Loadable Function]

These routines compute the integral

Chi(x) := Re[gamma˙E + log(x) + int˙0^x dt (cosh[t]-1)/t] ,

where gamma˙E is the Euler constant.

err contains an estimate of the absolute error in the value *y*.

This function is from the GNU Scientific Library, see http://www.gnu.org/software/gsl/ for documentation.

Øexpint˙3 -*- texinfo -*-

`y = expint_3 (x)` [Loadable Function]
`[y, err] = expint_3 (...)` [Loadable Function]

These routines compute the exponential integral Ei˙3(x) = int˙0^x dt exp(-t^3) for x >= 0.

err contains an estimate of the absolute error in the value *y*.

This function is from the GNU Scientific Library, see http://www.gnu.org/software/gsl/ for documentation.

ØSi -*- texinfo -*-

`y = Si (x)` [Loadable Function]
`[y, err] = Si (...)` [Loadable Function]

These routines compute the Sine integral Si(x) = int˙0^x dt sin(t)/t.

err contains an estimate of the absolute error in the value *y*.

This function is from the GNU Scientific Library, see http://www.gnu.org/software/gsl/ for documentation.

ØCi -*- texinfo -*-

`y = Ci (x)` [Loadable Function]
`[y, err] = Ci (...)` [Loadable Function]

These routines compute the Cosine integral Ci(x) = -int˙x^infty dt cos(t)/t for x > 0.

err contains an estimate of the absolute error in the value *y*.

This function is from the GNU Scientific Library, see http://www.gnu.org/software/gsl/ for documentation.

Øatanint -*- texinfo -*-

`y = atanint (x)` [Loadable Function]
`[y, err] = atanint (...)` [Loadable Function]

These routines compute the Arctangent integral AtanInt(x) = int˙0^x dt arctan(t)/t.

err contains an estimate of the absolute error in the value *y*.

This function is from the GNU Scientific Library, see http://www.gnu.org/software/gsl/ for documentation.

Øfermi˙dirac˙mhalf -*- texinfo -*-

`y = fermi_dirac_mhalf (x)` [Loadable Function]

`[y, err] = fermi_dirac_mhalf (...)` [Loadable Function]

These routines compute the complete Fermi-Dirac integral F˙{-1/2}(x).

err contains an estimate of the absolute error in the value *y*.

This function is from the GNU Scientific Library, see http://www.gnu.org/software/gsl/ for documentation.

Øfermi˙dirac˙half -*- texinfo -*-

`y = fermi_dirac_half (x)` [Loadable Function]

`[y, err] = fermi_dirac_half (...)` [Loadable Function]

These routines compute the complete Fermi-Dirac integral F˙{1/2}(x).

err contains an estimate of the absolute error in the value *y*.

This function is from the GNU Scientific Library, see http://www.gnu.org/software/gsl/ for documentation.

Øfermi˙dirac˙3half -*- texinfo -*-

`y = fermi_dirac_3half (x)` [Loadable Function]

`[y, err] = fermi_dirac_3half (...)` [Loadable Function]

These routines compute the complete Fermi-Dirac integral F˙{3/2}(x).

err contains an estimate of the absolute error in the value *y*.

This function is from the GNU Scientific Library, see http://www.gnu.org/software/gsl/ for documentation.

Øgamma˙gsl -*- texinfo -*-

`y = gamma_gsl (x)` [Loadable Function]

`[y, err] = gamma_gsl (...)` [Loadable Function]

These routines compute the Gamma function Gamma(x), subject to x not being a negative integer. The function is computed using the real Lanczos method. The maximum value of x such that Gamma(x) is not considered an overflow is given by the macro GSL˙SF˙GAMMA˙XMAX and is 171.0.

err contains an estimate of the absolute error in the value *y*.

This function is from the GNU Scientific Library, see http://www.gnu.org/software/gsl/ for documentation.

Ølngamma˙gsl -*- texinfo -*-

`y = lngamma_gsl (x)` [Loadable Function]

`[y, err] = lngamma_gsl (...)` [Loadable Function]

These routines compute the logarithm of the Gamma function, log(Gamma(x)), subject to x not a being negative integer. For x<0 the real part of log(Gamma(x)) is returned, which is equivalent to log(|Gamma(x)|). The function is computed using the real Lanczos method.

err contains an estimate of the absolute error in the value *y*.

This function is from the GNU Scientific Library, see http://www.gnu.org/software/gsl/ for documentation.

Øgammastar -*- texinfo -*-

y = gammastar (*x*) [Loadable Function]
[*y*, *err*] = gammastar (. . .) [Loadable Function]

These routines compute the regulated Gamma Function Gamma^*(x) for x > 0. The regulated gamma function is given by,

Gamma^*(x) = Gamma(x)/(sqrt{2pi} x^{(x-1/2)} exp(-x)) = (1 + (1/12x) + ...) for x to infty

and is a useful suggestion of Temme.

err contains an estimate of the absolute error in the value *y*.

This function is from the GNU Scientific Library, see http://www.gnu.org/software/gsl/ for documentation.

Øgammainv˙gsl -*- texinfo -*-

y = gammainv_gsl (*x*) [Loadable Function]
[*y*, *err*] = gammainv_gsl (. . .) [Loadable Function]

These routines compute the reciprocal of the gamma function, 1/Gamma(x) using the real Lanczos method.

err contains an estimate of the absolute error in the value *y*.

This function is from the GNU Scientific Library, see http://www.gnu.org/software/gsl/ for documentation.

Ølambert˙W0 -*- texinfo -*-

y = lambert_W0 (*x*) [Loadable Function]
[*y*, *err*] = lambert_W0 (. . .) [Loadable Function]

These compute the principal branch of the Lambert W function, W˙0(x).

Lambert's W functions, W(x), are defined to be solutions of the equation W(x) exp(W(x)) = x. This function has multiple branches for x < 0; however, it has only two real-valued branches. We define W˙0(x) to be the principal branch, where W > -1 for x < 0, and W˙{-1}(x) to be the other real branch, where W < -1 for x < 0.

err contains an estimate of the absolute error in the value *y*.

This function is from the GNU Scientific Library, see http://www.gnu.org/software/gsl/ for documentation.

Ølambert˙Wm1 -*- texinfo -*-

y = lambert_Wm1 (*x*) [Loadable Function]
[*y*, *err*] = lambert_Wm1 (. . .) [Loadable Function]

These compute the secondary real-valued branch of the Lambert W function, W˙{-1}(x).

Lambert's W functions, W(x), are defined to be solutions of the equation W(x) exp(W(x)) = x. This function has multiple branches for x < 0; however, it has only two real-valued branches. We define W˙0(x) to be the principal branch, where W > -1 for x < 0, and W˙{-1}(x) to be the other real branch, where W < -1 for x < 0.

err contains an estimate of the absolute error in the value *y*.

This function is from the GNU Scientific Library, see http://www.gnu.org/software/gsl/ for documentation.

Ølog˙1plusx -*- texinfo -*-

y = log_1plusx (x) [Loadable Function]
[y, err] = log_1plusx (. . .) [Loadable Function]

These routines compute log(1 + x) for x > -1 using an algorithm that is accurate for small x.

err contains an estimate of the absolute error in the value *y*.

This function is from the GNU Scientific Library, see http://www.gnu.org/software/gsl/ for documentation.

Ølog˙1plusx˙mx -*- texinfo -*-

y = log_1plusx_mx (x) [Loadable Function]
[y, err] = log_1plusx_mx (. . .) [Loadable Function]

These routines compute log(1 + x) - x for x > -1 using an algorithm that is accurate for small x.

err contains an estimate of the absolute error in the value *y*.

This function is from the GNU Scientific Library, see http://www.gnu.org/software/gsl/ for documentation.

Øpsi -*- texinfo -*-

y = psi (x) [Loadable Function]
[y, err] = psi (. . .) [Loadable Function]

These routines compute the digamma function psi(x) for general x, x e 0.

err contains an estimate of the absolute error in the value *y*.

This function is from the GNU Scientific Library, see http://www.gnu.org/software/gsl/ for documentation.

Øpsi˙1piy -*- texinfo -*-

y = psi_1piy (x) [Loadable Function]
[y, err] = psi_1piy (. . .) [Loadable Function]

These routines compute the real part of the digamma function on the line 1+i y, Re[psi(1 + i y)].

err contains an estimate of the absolute error in the value *y*.

This function is from the GNU Scientific Library, see http://www.gnu.org/software/gsl/ for documentation.

Øsynchrotron˙1 -*- texinfo -*-

y = synchrotron_1 (x) [Loadable Function]
[y, err] = synchrotron_1 (. . .) [Loadable Function]

These routines compute the first synchrotron function x int˙x^infty dt K˙{5/3}(t) for x >= 0.

err contains an estimate of the absolute error in the value *y*.

This function is from the GNU Scientific Library, see http://www.gnu.org/software/gsl/ for documentation.

Øsynchrotron˙2 -*- texinfo -*-

`y = synchrotron_2 (x)` [Loadable Function]
`[y, err] = synchrotron_2 (...)` [Loadable Function]

These routines compute the second synchrotron function x K˙{2/3}(x) for x >= 0.

err contains an estimate of the absolute error in the value *y*.

This function is from the GNU Scientific Library, see http://www.gnu.org/software/gsl/ for documentation.

Øtransport˙2 -*- texinfo -*-

`y = transport_2 (x)` [Loadable Function]
`[y, err] = transport_2 (...)` [Loadable Function]

These routines compute the transport function J(2,x).

The transport functions J(n,x) are defined by the integral representations J(n,x) := int˙0^x dt t^n e^t /(e^t - 1)^2.

err contains an estimate of the absolute error in the value *y*.

This function is from the GNU Scientific Library, see http://www.gnu.org/software/gsl/ for documentation.

Øtransport˙3 -*- texinfo -*-

`y = transport_3 (x)` [Loadable Function]
`[y, err] = transport_3 (...)` [Loadable Function]

These routines compute the transport function J(3,x).

The transport functions J(n,x) are defined by the integral representations J(n,x) := int˙0^x dt t^n e^t /(e^t - 1)^2.

err contains an estimate of the absolute error in the value *y*.

This function is from the GNU Scientific Library, see http://www.gnu.org/software/gsl/ for documentation.

Øtransport˙4 -*- texinfo -*-

`y = transport_4 (x)` [Loadable Function]
`[y, err] = transport_4 (...)` [Loadable Function]

These routines compute the transport function J(4,x).

The transport functions J(n,x) are defined by the integral representations J(n,x) := int˙0^x dt t^n e^t /(e^t - 1)^2.

err contains an estimate of the absolute error in the value *y*.

This function is from the GNU Scientific Library, see http://www.gnu.org/software/gsl/ for documentation.

Øtransport˙5 -*- texinfo -*-

`y = transport_5 (x)` [Loadable Function]
`[y, err] = transport_5 (...)` [Loadable Function]

These routines compute the transport function J(5,x).

The transport functions J(n,x) are defined by the integral representations J(n,x) := int˙0^x dt t^n e^t /(e^t - 1)^2.

err contains an estimate of the absolute error in the value *y*.

This function is from the GNU Scientific Library, see http://www.gnu.org/software/gsl/ for documentation.

Øsinc˙gsl -*- texinfo -*-

`y = sinc_gsl (x)` [Loadable Function]
`[y, err] = sinc_gsl (...)` [Loadable Function]

These routines compute sinc(x) = sin(pi x) / (pi x) for any value of x.

err contains an estimate of the absolute error in the value *y*.

This function is from the GNU Scientific Library, see http://www.gnu.org/software/gsl/ for documentation.

Ølnsinh -*- texinfo -*-

`y = lnsinh (x)` [Loadable Function]
`[y, err] = lnsinh (...)` [Loadable Function]

These routines compute log(sinh(x)) for x > 0.

err contains an estimate of the absolute error in the value *y*.

This function is from the GNU Scientific Library, see http://www.gnu.org/software/gsl/ for documentation.

Ølncosh -*- texinfo -*-

`y = lncosh (x)` [Loadable Function]
`[y, err] = lncosh (...)` [Loadable Function]

These routines compute log(cosh(x)) for any x.

err contains an estimate of the absolute error in the value *y*.

This function is from the GNU Scientific Library, see http://www.gnu.org/software/gsl/ for documentation.

Øzeta -*- texinfo -*-

`y = zeta (x)` [Loadable Function]
`[y, err] = zeta (...)` [Loadable Function]

These routines compute the Riemann zeta function zeta(s) for arbitrary s, s e 1.

The Riemann zeta function is defined by the infinite sum zeta(s) = sum˙{k=1}^infty k^{-s}.

err contains an estimate of the absolute error in the value *y*.

This function is from the GNU Scientific Library, see http://www.gnu.org/software/gsl/ for documentation.

Øeta -*- texinfo -*-

`y = eta (x)` [Loadable Function]
`[y, err] = eta (...)` [Loadable Function]

These routines compute the eta function eta(s) for arbitrary s.

The eta function is defined by eta(s) = (1-2^{1-s}) zeta(s).

err contains an estimate of the absolute error in the value *y*.

This function is from the GNU Scientific Library, see http://www.gnu.org/software/gsl/ for documentation.

Øbessel˙Jn -*- texinfo -*-

`y = bessel_Jn (n, x)` [Loadable Function]
`[y, err] = bessel_Jn (...)` [Loadable Function]

These routines compute the regular cylindrical Bessel function of order n, J˙n(x).

err contains an estimate of the absolute error in the value *y*.

This function is from the GNU Scientific Library, see http://www.gnu.org/software/gsl/ for documentation.

Øbessel˙Yn -*- texinfo -*-

`y = bessel_Yn (n, x)` [Loadable Function]
`[y, err] = bessel_Yn (...)` [Loadable Function]

These routines compute the irregular cylindrical Bessel function of order n, Y˙n(x), for x>0.

err contains an estimate of the absolute error in the value *y*.

This function is from the GNU Scientific Library, see http://www.gnu.org/software/gsl/ for documentation.

Øbessel˙In -*- texinfo -*-

`y = bessel_In (n, x)` [Loadable Function]
`[y, err] = bessel_In (...)` [Loadable Function]

These routines compute the regular modified cylindrical Bessel function of order n, I˙n(x).

err contains an estimate of the absolute error in the value *y*.

This function is from the GNU Scientific Library, see http://www.gnu.org/software/gsl/ for documentation.

Øbessel˙In˙scaled -*- texinfo -*-

`y = bessel_In_scaled (n, x)` [Loadable Function]
`[y, err] = bessel_In_scaled (...)` [Loadable Function]

These routines compute the scaled regular modified cylindrical Bessel function of order n, exp(-|x|) I˙n(x)

err contains an estimate of the absolute error in the value *y*.

This function is from the GNU Scientific Library, see http://www.gnu.org/software/gsl/ for documentation.

Øbessel˙Kn -*- texinfo -*-

`y = bessel_Kn (n, x)` [Loadable Function]
`[y, err] = bessel_Kn (...)` [Loadable Function]

These routines compute the irregular modified cylindrical Bessel function of order n, K˙n(x), for x > 0.

err contains an estimate of the absolute error in the value *y*.

This function is from the GNU Scientific Library, see http://www.gnu.org/software/gsl/ for documentation.

Øbessel˙Kn˙scaled -*- texinfo -*-

`y = bessel_Kn_scaled (n, x)` [Loadable Function]
`[y, err] = bessel_Kn_scaled (...)` [Loadable Function]

err contains an estimate of the absolute error in the value *y*.

This function is from the GNU Scientific Library, see http://www.gnu.org/software/gsl/ for documentation.

Øbessel˙jl -*- texinfo -*-

`y = bessel_jl (n, x)` [Loadable Function]
`[y, err] = bessel_jl (...)` [Loadable Function]

These routines compute the regular spherical Bessel function of order l, j˙l(x), for l >= 0 and x >= 0.

err contains an estimate of the absolute error in the value *y*.

This function is from the GNU Scientific Library, see http://www.gnu.org/software/gsl/ for documentation.

Øbessel˙yl -*- texinfo -*-

`y = bessel_yl (n, x)` [Loadable Function]
`[y, err] = bessel_yl (...)` [Loadable Function]

These routines compute the irregular spherical Bessel function of order l, y˙l(x), for l >= 0.

err contains an estimate of the absolute error in the value *y*.

This function is from the GNU Scientific Library, see http://www.gnu.org/software/gsl/ for documentation.

Øbessel˙il˙scaled -*- texinfo -*-

`y = bessel_il_scaled (n, x)` [Loadable Function]
`[y, err] = bessel_il_scaled (...)` [Loadable Function]

These routines compute the scaled regular modified spherical Bessel function of order l, exp(-|x|) i˙l(x)

err contains an estimate of the absolute error in the value *y*.

This function is from the GNU Scientific Library, see http://www.gnu.org/software/gsl/ for documentation.

Øbessel˙kl˙scaled -*- texinfo -*-

y = **bessel_kl_scaled** (*n*, *x*) [Loadable Function]
[*y*, *err*] = **bessel_kl_scaled** (...) [Loadable Function]

These routines compute the scaled irregular modified spherical Bessel function of order l, exp(x) k˙l(x), for x>0.

err contains an estimate of the absolute error in the value *y*.

This function is from the GNU Scientific Library, see http://www.gnu.org/software/gsl/ for documentation.

Øexprel˙n -*- texinfo -*-

y = **exprel_n** (*n*, *x*) [Loadable Function]
[*y*, *err*] = **exprel_n** (...) [Loadable Function]

These routines compute the N-relative exponential, which is the n-th generalization of the functions gsl˙sf˙exprel and gsl˙sf˙exprel2. The N-relative exponential is given by,

exprel˙N(x) = N!/x^N (exp(x) - sum˙{k=0}^{N-1} x^k/k!) = 1 + x/(N+1) + x^2/((N+1)(N+2)) + ... = 1F1 (1,1+N,x)

err contains an estimate of the absolute error in the value *y*.

This function is from the GNU Scientific Library, see http://www.gnu.org/software/gsl/ for documentation.

Øfermi˙dirac˙int -*- texinfo -*-

y = **fermi_dirac_int** (*n*, *x*) [Loadable Function]
[*y*, *err*] = **fermi_dirac_int** (...) [Loadable Function]

These routines compute the complete Fermi-Dirac integral with an integer index of j, F˙j(x) = (1/Gamma(j+1)) int˙0^infty dt (t^j /(exp(t-x)+1)).

err contains an estimate of the absolute error in the value *y*.

This function is from the GNU Scientific Library, see http://www.gnu.org/software/gsl/ for documentation.

Øtaylorcoeff -*- texinfo -*-

y = **taylorcoeff** (*n*, *x*) [Loadable Function]
[*y*, *err*] = **taylorcoeff** (...) [Loadable Function]

These routines compute the Taylor coefficient x^n / n! for x >= 0, n >= 0.

err contains an estimate of the absolute error in the value *y*.

This function is from the GNU Scientific Library, see http://www.gnu.org/software/gsl/ for documentation.

Ølegendre˙Pl -*- texinfo -*-

y = **legendre_Pl** (*n*, *x*) [Loadable Function]
[*y*, *err*] = **legendre_Pl** (...) [Loadable Function]

These functions evaluate the Legendre polynomial P˙l(x) for a specific value of l, x subject to l >= 0, |x| <= 1

err contains an estimate of the absolute error in the value *y*.

This function is from the GNU Scientific Library, see http://www.gnu.org/software/gsl/ for documentation.

Ølegendre˙Ql -*- texinfo -*-

y = legendre_Ql (*n*, *x*) [Loadable Function]
[*y*, *err*] = legendre_Ql (...) [Loadable Function]

These routines compute the Legendre function Q˙l(x) for x > -1, x != 1 and l >= 0.

err contains an estimate of the absolute error in the value *y*.

This function is from the GNU Scientific Library, see http://www.gnu.org/software/gsl/ for documentation.

Øpsi˙n -*- texinfo -*-

y = psi_n (*n*, *x*) [Loadable Function]
[*y*, *err*] = psi_n (...) [Loadable Function]

These routines compute the polygamma function psi^{(m)}(x) for m >= 0, x > 0.

err contains an estimate of the absolute error in the value *y*.

This function is from the GNU Scientific Library, see http://www.gnu.org/software/gsl/ for documentation.

Øbessel˙Jnu -*- texinfo -*-

z = bessel_Jnu (*x*, *y*) [Loadable Function]
[*z*, *err*] = bessel_Jnu (...) [Loadable Function]

These routines compute the regular cylindrical Bessel function of fractional order nu, J˙u(x).

err contains an estimate of the absolute error in the value *z*.

This function is from the GNU Scientific Library, see http://www.gnu.org/software/gsl/ for documentation.

Øbessel˙Ynu -*- texinfo -*-

z = bessel_Ynu (*x*, *y*) [Loadable Function]
[*z*, *err*] = bessel_Ynu (...) [Loadable Function]

These routines compute the irregular cylindrical Bessel function of fractional order nu, Y˙u(x).

err contains an estimate of the absolute error in the value *z*.

This function is from the GNU Scientific Library, see http://www.gnu.org/software/gsl/ for documentation.

Øbessel˙Inu -*- texinfo -*-

z = bessel_Inu (*x*, *y*) [Loadable Function]
[*z*, *err*] = bessel_Inu (...) [Loadable Function]

These routines compute the regular modified Bessel function of fractional order nu, I˙u(x) for x>0, u>0.

err contains an estimate of the absolute error in the value *z*.

This function is from the GNU Scientific Library, see http://www.gnu.org/software/gsl/ for documentation.

Øbessel˙Inu˙scaled -*- texinfo -*-

z = **bessel_Inu_scaled** (*x*, *y*) [Loadable Function]
[*z*, *err*] = **bessel_Inu_scaled** (. . .) [Loadable Function]

These routines compute the scaled regular modified Bessel function of fractional order nu, exp(-|x|)I˙u(x) for x>0, u>0.

err contains an estimate of the absolute error in the value *z*.

This function is from the GNU Scientific Library, see http://www.gnu.org/software/gsl/ for documentation.

Øbessel˙Knu -*- texinfo -*-

z = **bessel_Knu** (*x*, *y*) [Loadable Function]
[*z*, *err*] = **bessel_Knu** (. . .) [Loadable Function]

These routines compute the irregular modified Bessel function of fractional order nu, K˙u(x) for x>0, u>0.

err contains an estimate of the absolute error in the value *z*.

This function is from the GNU Scientific Library, see http://www.gnu.org/software/gsl/ for documentation.

Øbessel˙lnKnu -*- texinfo -*-

z = **bessel_lnKnu** (*x*, *y*) [Loadable Function]
[*z*, *err*] = **bessel_lnKnu** (. . .) [Loadable Function]

These routines compute the logarithm of the irregular modified Bessel function of fractional order nu, ln(K˙u(x)) for x>0, u>0.

err contains an estimate of the absolute error in the value *z*.

This function is from the GNU Scientific Library, see http://www.gnu.org/software/gsl/ for documentation.

Øbessel˙Knu˙scaled -*- texinfo -*-

z = **bessel_Knu_scaled** (*x*, *y*) [Loadable Function]
[*z*, *err*] = **bessel_Knu_scaled** (. . .) [Loadable Function]

These routines compute the scaled irregular modified Bessel function of fractional order nu, exp(+|x|) K˙u(x) for x>0, u>0.

err contains an estimate of the absolute error in the value *z*.

This function is from the GNU Scientific Library, see http://www.gnu.org/software/gsl/ for documentation.

Øexp˙mult -*- texinfo -*-

`z = exp_mult (x, y)` [Loadable Function]
`[z, err] = exp_mult (...)` [Loadable Function]

These routines exponentiate x and multiply by the factor y to return the product y exp(x).

err contains an estimate of the absolute error in the value *z*.

This function is from the GNU Scientific Library, see http://www.gnu.org/software/gsl/ for documentation.

Øfermi˙dirac˙inc˙0 -*- texinfo -*-

`z = fermi_dirac_inc_0 (x, y)` [Loadable Function]
`[z, err] = fermi_dirac_inc_0 (...)` [Loadable Function]

These routines compute the incomplete Fermi-Dirac integral with an index of zero, F˙0(x,b) = ln(1 + e^{b-x}) - (b-x).

err contains an estimate of the absolute error in the value *z*.

This function is from the GNU Scientific Library, see http://www.gnu.org/software/gsl/ for documentation.

Øpoch -*- texinfo -*-

`z = poch (x, y)` [Loadable Function]
`[z, err] = poch (...)` [Loadable Function]

These routines compute the Pochhammer symbol

(a)˙x := Gamma(a + x)/Gamma(a),

subject to a and a+x not being negative integers. The Pochhammer symbol is also known as the Apell symbol.

err contains an estimate of the absolute error in the value *z*.

This function is from the GNU Scientific Library, see http://www.gnu.org/software/gsl/ for documentation.

Ølnpoch -*- texinfo -*-

`z = lnpoch (x, y)` [Loadable Function]
`[z, err] = lnpoch (...)` [Loadable Function]

These routines compute the logarithm of the Pochhammer symbol, log((a)˙x) = log(Gamma(a + x)/Gamma(a)) for a > 0, a+x > 0.

err contains an estimate of the absolute error in the value *z*.

This function is from the GNU Scientific Library, see http://www.gnu.org/software/gsl/ for documentation.

Øpochrel -*- texinfo -*-

`z = pochrel (x, y)` [Loadable Function]
`[z, err] = pochrel (...)` [Loadable Function]

These routines compute the relative Pochhammer symbol ((a,x) - 1)/x where (a,x) = (a)˙x := Gamma(a + x)/Gamma(a).

err contains an estimate of the absolute error in the value *z*.

This function is from the GNU Scientific Library, see http://www.gnu.org/software/gsl/ for documentation.

Øgamma˙inc˙Q -*- texinfo -*-

`z = gamma_inc_Q (x, y)` [Loadable Function]
`[z, err] = gamma_inc_Q (...)` [Loadable Function]

These routines compute the normalized incomplete Gamma Function Q(a,x) = 1/Gamma(a) int˙xinfty dt t^{a-1} exp(-t) for a > 0, x >= 0.

err contains an estimate of the absolute error in the value *z*.

This function is from the GNU Scientific Library, see http://www.gnu.org/software/gsl/ for documentation.

Øgamma˙inc˙P -*- texinfo -*-

`z = gamma_inc_P (x, y)` [Loadable Function]
`[z, err] = gamma_inc_P (...)` [Loadable Function]

These routines compute the complementary normalized incomplete Gamma Function P(a,x) = 1/Gamma(a) int˙0^x dt t^{a-1} exp(-t) for a > 0, x >= 0.

err contains an estimate of the absolute error in the value *z*.

This function is from the GNU Scientific Library, see http://www.gnu.org/software/gsl/ for documentation.

Øgamma˙inc -*- texinfo -*-

`z = gamma_inc (x, y)` [Loadable Function]
`[z, err] = gamma_inc (...)` [Loadable Function]

These functions compute the incomplete Gamma Function the normalization factor included in the previously defined functions: Gamma(a,x) = int˙xinfty dt t^{a-1} exp(-t) for a real and x >= 0.

err contains an estimate of the absolute error in the value *z*.

This function is from the GNU Scientific Library, see http://www.gnu.org/software/gsl/ for documentation.

Øbeta˙gsl -*- texinfo -*-

`z = beta_gsl (x, y)` [Loadable Function]
`[z, err] = beta_gsl (...)` [Loadable Function]

These routines compute the Beta Function, B(a,b) = Gamma(a)Gamma(b)/Gamma(a+b) for a > 0, b > 0.

err contains an estimate of the absolute error in the value *z*.

This function is from the GNU Scientific Library, see http://www.gnu.org/software/gsl/ for documentation.

Ølnbeta -*- texinfo -*-

`z = lnbeta (x, y)` [Loadable Function]
`[z, err] = lnbeta (...)` [Loadable Function]

These routines compute the logarithm of the Beta Function, log(B(a,b)) for a > 0, b > 0.

err contains an estimate of the absolute error in the value *z*.

This function is from the GNU Scientific Library, see http://www.gnu.org/software/gsl/ for documentation.

Øhyperg˙0F1 -*- texinfo -*-

`z = hyperg_0F1 (x, y)` [Loadable Function]
`[z, err] = hyperg_0F1 (...)` [Loadable Function]

These routines compute the hypergeometric function 0F1(c,x).

err contains an estimate of the absolute error in the value *z*.

This function is from the GNU Scientific Library, see `http://www.gnu.org/software/gsl/` for documentation.

ØconicalP˙half -*- texinfo -*-

`z = conicalP_half (x, y)` [Loadable Function]
`[z, err] = conicalP_half (...)` [Loadable Function]

These routines compute the irregular Spherical Conical Function P^{1/2}˙{-1/2 + i lambda}(x) for x > -1.

err contains an estimate of the absolute error in the value *z*.

This function is from the GNU Scientific Library, see `http://www.gnu.org/software/gsl/` for documentation.

ØconicalP˙mhalf -*- texinfo -*-

`z = conicalP_mhalf (x, y)` [Loadable Function]
`[z, err] = conicalP_mhalf (...)` [Loadable Function]

These routines compute the regular Spherical Conical Function P^{-1/2}˙{-1/2 + i lambda}(x) for x > -1.

err contains an estimate of the absolute error in the value *z*.

This function is from the GNU Scientific Library, see `http://www.gnu.org/software/gsl/` for documentation.

ØconicalP˙0 -*- texinfo -*-

`z = conicalP_0 (x, y)` [Loadable Function]
`[z, err] = conicalP_0 (...)` [Loadable Function]

These routines compute the conical function P^0˙{-1/2 + i lambda}(x) for x > -1.

err contains an estimate of the absolute error in the value *z*.

This function is from the GNU Scientific Library, see `http://www.gnu.org/software/gsl/` for documentation.

ØconicalP˙1 -*- texinfo -*-

`z = conicalP_1 (x, y)` [Loadable Function]
`[z, err] = conicalP_1 (...)` [Loadable Function]

These routines compute the conical function P^1˙{-1/2 + i lambda}(x) for x > -1.

err contains an estimate of the absolute error in the value *z*.

This function is from the GNU Scientific Library, see `http://www.gnu.org/software/gsl/` for documentation.

Øhzeta -*- texinfo -*-

`z = hzeta (x, y)` [Loadable Function]
`[z, err] = hzeta (...)` [Loadable Function]

These routines compute the Hurwitz zeta function zeta(s,q) for s > 1, q > 0.

err contains an estimate of the absolute error in the value *z*.

This function is from the GNU Scientific Library, see http://www.gnu.org/software/gsl/ for documentation.

Øairy˙Ai -*- texinfo -*-

`y = airy_Ai (x, mode)` [Loadable Function]
`[y, err] = airy_Ai (...)` [Loadable Function]

These routines compute the Airy function Ai(x) with an accuracy specified by mode.

The second argument *mode* must be an integer corresponding to

0 = GSL˙PREC˙DOUBLE
: Double-precision, a relative accuracy of approximately `2 * 10^-16`.

1 = GSL˙PREC˙SINGLE
: Single-precision, a relative accuracy of approximately `10^-7`.

2 = GSL˙PREC˙APPROX
: Approximate values, a relative accuracy of approximately `5 * 10^-4`.

err contains an estimate of the absolute error in the value *y*.

This function is from the GNU Scientific Library, see http://www.gnu.org/software/gsl/ for documentation.

Øairy˙Bi -*- texinfo -*-

`y = airy_Bi (x, mode)` [Loadable Function]
`[y, err] = airy_Bi (...)` [Loadable Function]

These routines compute the Airy function Bi(x) with an accuracy specified by mode.

The second argument *mode* must be an integer corresponding to

0 = GSL˙PREC˙DOUBLE
: Double-precision, a relative accuracy of approximately `2 * 10^-16`.

1 = GSL˙PREC˙SINGLE
: Single-precision, a relative accuracy of approximately `10^-7`.

2 = GSL˙PREC˙APPROX
: Approximate values, a relative accuracy of approximately `5 * 10^-4`.

err contains an estimate of the absolute error in the value *y*.

This function is from the GNU Scientific Library, see http://www.gnu.org/software/gsl/ for documentation.

Øairy˙Ai˙scaled -*- texinfo -*-

`y = airy_Ai_scaled (x, mode)` [Loadable Function]
`[y, err] = airy_Ai_scaled (...)` [Loadable Function]

These routines compute a scaled version of the Airy function S˙A(x) Ai(x). For x>0 the scaling factor S˙A(x) is exp(+(2/3) x^(3/2)), and is 1 for x<0.

The second argument *mode* must be an integer corresponding to

0 = GSL˙PREC˙DOUBLE
: Double-precision, a relative accuracy of approximately `2 * 10^-16`.

1 = GSL˙PREC˙SINGLE
: Single-precision, a relative accuracy of approximately `10^-7`.

2 = GSL˙PREC˙APPROX
: Approximate values, a relative accuracy of approximately `5 * 10^-4`.

err contains an estimate of the absolute error in the value *y*.

This function is from the GNU Scientific Library, see http://www.gnu.org/software/gsl/ for documentation.

Øairy˙Bi˙scaled -*- texinfo -*-

`y = airy_Bi_scaled (x, mode)` [Loadable Function]
`[y, err] = airy_Bi_scaled (...)` [Loadable Function]

These routines compute a scaled version of the Airy function S˙B(x) Bi(x). For x>0 the scaling factor S˙B(x) is exp(-(2/3) x^(3/2)), and is 1 for x<0.

The second argument *mode* must be an integer corresponding to

0 = GSL˙PREC˙DOUBLE
: Double-precision, a relative accuracy of approximately `2 * 10^-16`.

1 = GSL˙PREC˙SINGLE
: Single-precision, a relative accuracy of approximately `10^-7`.

2 = GSL˙PREC˙APPROX
: Approximate values, a relative accuracy of approximately `5 * 10^-4`.

err contains an estimate of the absolute error in the value *y*.

This function is from the GNU Scientific Library, see http://www.gnu.org/software/gsl/ for documentation.

Øairy˙Ai˙deriv -*- texinfo -*-

`y = airy_Ai_deriv (x, mode)` [Loadable Function]
`[y, err] = airy_Ai_deriv (...)` [Loadable Function]

These routines compute the Airy function derivative Ai'(x) with an accuracy specified by mode.

The second argument *mode* must be an integer corresponding to

0 = GSL˙PREC˙DOUBLE
: Double-precision, a relative accuracy of approximately `2 * 10^-16`.

1 = GSL˙PREC˙SINGLE
: Single-precision, a relative accuracy of approximately `10^-7`.

2 = GSL˙PREC˙APPROX
: Approximate values, a relative accuracy of approximately `5 * 10^-4`.

err contains an estimate of the absolute error in the value *y*.

This function is from the GNU Scientific Library, see http://www.gnu.org/software/gsl/ for documentation.

Øairy˙Bi˙deriv -*- texinfo -*-

y = **airy_Bi_deriv** (*x*, *mode*) [Loadable Function]
[*y*, *err*] = **airy_Bi_deriv** (...) [Loadable Function]

These routines compute the Airy function derivative Bi'(x) with an accuracy specified by mode.

The second argument *mode* must be an integer corresponding to

0 = GSL˙PREC˙DOUBLE
: Double-precision, a relative accuracy of approximately `2 * 10^-16`.

1 = GSL˙PREC˙SINGLE
: Single-precision, a relative accuracy of approximately `10^-7`.

2 = GSL˙PREC˙APPROX
: Approximate values, a relative accuracy of approximately `5 * 10^-4`.

err contains an estimate of the absolute error in the value *y*.

This function is from the GNU Scientific Library, see http://www.gnu.org/software/gsl/ for documentation.

Øairy˙Ai˙deriv˙scaled -*- texinfo -*-

y = **airy_Ai_deriv_scaled** (*x*, *mode*) [Loadable Function]
[*y*, *err*] = **airy_Ai_deriv_scaled** (...) [Loadable Function]

These routines compute the derivative of the scaled Airy function S˙A(x) Ai(x).

The second argument *mode* must be an integer corresponding to

0 = GSL˙PREC˙DOUBLE
: Double-precision, a relative accuracy of approximately `2 * 10^-16`.

1 = GSL˙PREC˙SINGLE
: Single-precision, a relative accuracy of approximately `10^-7`.

2 = GSL˙PREC˙APPROX
: Approximate values, a relative accuracy of approximately `5 * 10^-4`.

err contains an estimate of the absolute error in the value *y*.

This function is from the GNU Scientific Library, see http://www.gnu.org/software/gsl/ for documentation.

Øairy˙Bi˙deriv˙scaled -*- texinfo -*-

`y = airy_Bi_deriv_scaled (x, mode)` [Loadable Function]
`[y, err] = airy_Bi_deriv_scaled (...)` [Loadable Function]

These routines compute the derivative of the scaled Airy function S˙B(x) Bi(x).

The second argument *mode* must be an integer corresponding to

0 = GSL˙PREC˙DOUBLE
: Double-precision, a relative accuracy of approximately `2 * 10^-16`.

1 = GSL˙PREC˙SINGLE
: Single-precision, a relative accuracy of approximately `10^-7`.

2 = GSL˙PREC˙APPROX
: Approximate values, a relative accuracy of approximately `5 * 10^-4`.

err contains an estimate of the absolute error in the value *y*.

This function is from the GNU Scientific Library, see http://www.gnu.org/software/gsl/ for documentation.

Øellint˙Kcomp -*- texinfo -*-

`y = ellint_Kcomp (x, mode)` [Loadable Function]
`[y, err] = ellint_Kcomp (...)` [Loadable Function]

These routines compute the complete elliptic integral K(k)

$$K(k) = \int_0^{\pi/2} \frac{dt}{\sqrt{(1 - k^2 \sin^2(t))}}$$

The notation used here is based on Carlson, *Numerische Mathematik* 33 (1979) and differs slightly from that used by Abramowitz & Stegun, where the functions are given in terms of the parameter $m = k^2$.

The second argument *mode* must be an integer corresponding to

0 = GSL˙PREC˙DOUBLE
: Double-precision, a relative accuracy of approximately `2 * 10^-16`.

1 = GSL˙PREC˙SINGLE
: Single-precision, a relative accuracy of approximately `10^-7`.

2 = GSL˙PREC˙APPROX
: Approximate values, a relative accuracy of approximately `5 * 10^-4`.

err contains an estimate of the absolute error in the value *y*.

This function is from the GNU Scientific Library, see http://www.gnu.org/software/gsl/ for documentation.

Øellint˙Ecomp -*- texinfo -*-

`y = ellint_Ecomp (x, mode)` [Loadable Function]
`[y, err] = ellint_Ecomp (...)` [Loadable Function]

These routines compute the complete elliptic integral E(k) to the accuracy specified by the mode variable mode.

$$E(k) = \int_0^{\pi/2} \sqrt{(1 - k^2 \sin^2(t))} dt$$

The notation used here is based on Carlson, *Numerische Mathematik* 33 (1979) and differs slightly from that used by Abramowitz & Stegun, where the functions are given in terms of the parameter $m = k^2$.

The second argument *mode* must be an integer corresponding to

0 = GSL˙PREC˙DOUBLE
: Double-precision, a relative accuracy of approximately `2 * 10^-16`.

1 = GSL˙PREC˙SINGLE
: Single-precision, a relative accuracy of approximately `10^-7`.

2 = GSL˙PREC˙APPROX
: Approximate values, a relative accuracy of approximately `5 * 10^-4`.

err contains an estimate of the absolute error in the value *y*.

This function is from the GNU Scientific Library, see `http://www.gnu.org/software/gsl/` for documentation.

Øairy˙zero˙Ai -*- texinfo -*-

`y = airy_zero_Ai (n)` [Loadable Function]
`[y, err] = airy_zero_Ai (...)` [Loadable Function]

These routines compute the location of the s-th zero of the Airy function Ai(x).

err contains an estimate of the absolute error in the value *y*.

This function is from the GNU Scientific Library, see `http://www.gnu.org/software/gsl/` for documentation.

Øairy˙zero˙Bi -*- texinfo -*-

`y = airy_zero_Bi (n)` [Loadable Function]
`[y, err] = airy_zero_Bi (...)` [Loadable Function]

These routines compute the location of the s-th zero of the Airy function Bi(x).

err contains an estimate of the absolute error in the value *y*.

This function is from the GNU Scientific Library, see `http://www.gnu.org/software/gsl/` for documentation.

Øairy˙zero˙Ai˙deriv -*- texinfo -*-

`y = airy_zero_Ai_deriv (n)` [Loadable Function]
`[y, err] = airy_zero_Ai_deriv (...)` [Loadable Function]

These routines compute the location of the s-th zero of the Airy function derivative Ai(x).

err contains an estimate of the absolute error in the value *y*.

This function is from the GNU Scientific Library, see `http://www.gnu.org/software/gsl/` for documentation.

Øairy˙zero˙Bi˙deriv -*- texinfo -*-

y = `airy_zero_Bi_deriv` (*n*) [Loadable Function]
[*y*, *err*] = `airy_zero_Bi_deriv` (...) [Loadable Function]

These routines compute the location of the s-th zero of the Airy function derivative Bi(x).

err contains an estimate of the absolute error in the value *y*.

This function is from the GNU Scientific Library, see http://www.gnu.org/software/gsl/ for documentation.

Øbessel˙zero˙J0 -*- texinfo -*-

y = `bessel_zero_J0` (*n*) [Loadable Function]
[*y*, *err*] = `bessel_zero_J0` (...) [Loadable Function]

These routines compute the location of the s-th positive zero of the Bessel function J˙0(x).

err contains an estimate of the absolute error in the value *y*.

This function is from the GNU Scientific Library, see http://www.gnu.org/software/gsl/ for documentation.

Øbessel˙zero˙J1 -*- texinfo -*-

y = `bessel_zero_J1` (*n*) [Loadable Function]
[*y*, *err*] = `bessel_zero_J1` (...) [Loadable Function]

These routines compute the location of the s-th positive zero of the Bessel function J˙1(x).

err contains an estimate of the absolute error in the value *y*.

This function is from the GNU Scientific Library, see http://www.gnu.org/software/gsl/ for documentation.

Øpsi˙1˙int -*- texinfo -*-

y = `psi_1_int` (*n*) [Loadable Function]
[*y*, *err*] = `psi_1_int` (...) [Loadable Function]

These routines compute the Trigamma function psi(n) for positive integer n.

err contains an estimate of the absolute error in the value *y*.

This function is from the GNU Scientific Library, see http://www.gnu.org/software/gsl/ for documentation.

Øzeta˙int -*- texinfo -*-

y = `zeta_int` (*n*) [Loadable Function]
[*y*, *err*] = `zeta_int` (...) [Loadable Function]

These routines compute the Riemann zeta function zeta(n) for integer n, n e 1.

err contains an estimate of the absolute error in the value *y*.

This function is from the GNU Scientific Library, see http://www.gnu.org/software/gsl/ for documentation.

Øeta˙int -*- texinfo -*-

`y = eta_int (n)` [Loadable Function]
`[y, err] = eta_int (...)` [Loadable Function]

These routines compute the eta function eta(n) for integer n.

err contains an estimate of the absolute error in the value *y*.

This function is from the GNU Scientific Library, see http://www.gnu.org/software/gsl/ for documentation.

Ølegendre˙Plm -*- texinfo -*-

`y = legendre_Plm (n, m, x)` [Loadable Function]
`[y, err] = legendre_Plm (...)` [Loadable Function]

These routines compute the associated Legendre polynomial P˙l^m(x) for m >= 0, l >= m, |x| <= 1.

err contains an estimate of the absolute error in the value *y*.

This function is from the GNU Scientific Library, see http://www.gnu.org/software/gsl/ for documentation.

Ølegendre˙sphPlm -*- texinfo -*-

`y = legendre_sphPlm (n, m, x)` [Loadable Function]
`[y, err] = legendre_sphPlm (...)` [Loadable Function]

These routines compute the normalized associated Legendre polynomial $sqrt{(2l+1)/(4pi)} sqrt{(l-m)!/(l+m)!} P˙l^m(x)$ suitable for use in spherical harmonics. The parameters must satisfy m >= 0, l >= m, |x| <= 1. Theses routines avoid the overflows that occur for the standard normalization of P˙l^m(x).

err contains an estimate of the absolute error in the value *y*.

This function is from the GNU Scientific Library, see http://www.gnu.org/software/gsl/ for documentation.

Øhyperg˙U -*- texinfo -*-

`out = hyperg_U (x0, x1, x2)` [Loadable Function]
`[out, err] = hyperg_U (...)` [Loadable Function]

Secondary Confluent Hypergoemetric U function A&E 13.1.3 All input are double as is the output.

err contains an estimate of the absolute error in the value *out*.a.

This function is from the GNU Scientific Library, see http://www.gnu.org/software/gsl/ for documentation.

Øhyperg˙1F1 -*- texinfo -*-

`out = hyperg_1F1 (x0, x1, x2)` [Loadable Function]
`[out, err] = hyperg_1F1 (...)` [Loadable Function]

Primary Confluent Hypergoemetric U function A&E 13.1.3 All inputs are double as is the output.

err contains an estimate of the absolute error in the value *out*.a.

This function is from the GNU Scientific Library, see http://www.gnu.org/software/gsl/ for documentation.
